# The transcription factor AREB1 regulates primary metabolic pathways in tomato fruits

**DOI:** 10.1093/jxb/eru114

**Published:** 2014-03-22

**Authors:** Adriana Bastías, Mónica Yañez, Sonia Osorio, Vicent Arbona, Aurelio Gómez-Cadenas, Alisdair R. Fernie, José A. Casaretto

**Affiliations:** ^1^Instituto de Biología Vegetal y Biotecnología, Universidad de Talca, 2 Norte 685, Talca, Chile; ^2^Max-Planck Institute of Molecular Plant Physiology, Am Muehlenberg 1, D-14476 Potsdam, Germany; ^3^Departament de Ciències Agràries i del Medi Natural, Universitat Jaume I, Campus Riu Sec, 12071 Castelló de la Plana, Spain

**Keywords:** Abscisic acid, AREB, fruit development, fruit ripening, metabolism, tomato.

## Abstract

Tomato fruit development is regulated both by the action of plant hormones and by tight genetic control. Recent studies suggest that abscisic acid (ABA) signalling may affect different aspects of fruit maturation. Previously, it was shown that SlAREB1, an ABA-regulated transcription factor involved in stress-induced responses, is expressed in seeds and in fruit tissues in tomato. Here, the role of SlAREB1 in regulating the expression of genes relevant for primary metabolic pathways and affecting the metabolic profile of the fruit was investigated using transgenic tomato lines. Metabolite profiling using gas chromatography–time of flight mass spectrometry (GC-TOF-MS) and non-targeted liquid chromatography–mass spectrometry (LC-MS) was performed on pericarp tissue from fruits harvested at three stages of fruit development. Principal component analysis of the data could distinguish the metabolite profiles of non-transgenic fruits from those that overexpress and down-regulate *SlAREB1*. Overexpression of *SlAREB1* resulted in increased content of organic acids, hexoses, hexose-phosphates, and amino acids in immature green, mature green, and red ripe fruits, and these modifications correlated with the up-regulation of enzyme-encoding genes involved in primary carbohydrate and amino acid metabolism. A non-targeted LC-MS analysis indicated that the composition of secondary metabolites is also affected in transgenic lines. In addition, gene expression data revealed that some genes associated with fruit ripening are also up-regulated in *SlAREB1*-overexpressing lines compared with wild-type and antisense lines. Taken together, the results suggest that SlAREB1 participates in the regulation of the metabolic programming that takes place during fruit ripening and that may explain part of the role of ABA in fruit development in tomato.

## Summary

Metabolite contents and expression of genes of primary metabolism are described in tomato fruits with different *SlAREB1* expression levels. Participation of this transcription factor and abscisic acid signalling in metabolic programming during fruit ripening is suggested.

## Introduction

Tomato is not only the most widespread and the most important horticultural crop worldwide but also is the most widely used model to study different aspects of fruit development. Auxin, cytokinin, gibberellins, abscisic acid (ABA), and ethylene modulate growth and several changes during fruit development, and thus are considered essential for the adequate completion of every stage of development (Ozga and Rienecke, 2003). In addition, new roles for other growth regulators such as polyamines, salicylic acid, jasmonic acid, and brassinosteroids in the development and the maturation of the fruit have been identified (Srivastava and Handa, 2005). In spite of extensive studies on the effects of hormones in plant growth and development, their mode of action at the molecular level in fruit development is still far from being understood. Only in recent years have some molecular components involved in the hormonal control during fruit development emerged (Osorio *et al.*, 2013).

One important aspect of fruit development is the modulation and regulation of its metabolism. Changes in sugars, organic acids, and secondary metabolites are very characteristic. During fruit development, the concentration of carbohydrates, amino acids, and organic acids diminishes immediately after fruit setting and partially recovers during or after fruit ripening (Carrari *et al.*, 2006). Quantification of a broad range of metabolites has been performed through metabolic profiling during fruit development. Transcriptomic and proteomic approaches have also been used to identify genetic networks of specific processes during fruit growth, such as fruit setting, early fruit tissue specialization, development of tomato fruit surface tissues, and ripening (Alba *et al.*, 2005; Rocco *et al.*, 2006; Mintz-Oron *et al.*, 2008; Vriezen *et al.*, 2008; Osorio *et al.*, 2011). These analyses have revealed a clear participation of enzymes, regulatory proteins, and hormonal signals in both primary and secondary metabolism. However, research on a link between hormonal signals that participate during this transition period and the control of gene expression involved in the modulation of metabolism is still ongoing.

Among plant hormones, ABA is well known as a key signal in response to different stress conditions. ABA content increases during maturation of both climacteric and non-climacteric fruits (McGlasson and Adato, 1976; Wang *et al.*, 2013). In tomato, ABA accumulates just prior the production of ethylene and has been suggested to be required for normal initiation of tomato fruit ripening, while ethylene functions in later stages of that process (Zhang *et al.*, 2009). Expression of core components of ABA signal perception, such as members of the *PYL*, *PP2C*, and *SnRK2* gene families, was described in tomato fruit (Sun *et al.*, 2011). Analysis of tomato databases revealed that genes encoding transcription factors that participate in ABA signalling are also expressed during fruit development. One of these factors, SlAREB1, belongs to a group of bZIP transcription factors known as ABA-response element-binding factors (AREBs) and are part of a major ABA-dependant signalling pathway that regulates gene expression in response to stress (Orellana *et al.*, 2010). Overexpression of *SlAREB1* in tomato resulted in increased tolerance to salt and water stress (Orellana *et al.*, 2010) as well as changes in the accumulation of organic acids and expression of genes encoding enzymes involved in their synthesis, suggesting a possible role for this transcription factor in the regulation of fruit organoleptic properties (Bastías *et al.*, 2011). In vegetative tissues, abiotic stress induces changes in metabolism, including sugars and amino acids (Urano *et al.*, 2009). In tomato, such conditions can also improve some aspects of fruit quality (Balibrea *et al.*, 2000; Yin *et al.*, 2009). For example, quantitive analyses have revealed that the content of some amino acids and sugars increases throughout fruit development in response to salt stress, whereas organic acid contents do not change significantly (Yin *et al*., 2009, 2010).

During fruit development, several metabolic pathways seem to be under transcriptional regulation controlled by different transcription factors (Rohrmann *et al.*, 2011). This work outlines the metabolic profiles of fruits with up- and down-regulation of the ABA-regulated factor *SlAREB1* determined by gas chromatography–time of flight mass spectrometry (GC-TOF-MS) and the expression of genes relevant for primary metabolic pathways. The results indicate that SlAREB1 affects the content of amino acids, organic acids, and sugar derivatives most probably by regulating the expression of enzyme-encoding genes involved in primary carbohydrate and amino acid metabolic pathways, suggesting that this transcription factor mediates an ABA signal that may be relevant for the regulation of the metabolic programming that takes place during fruit ripening in tomato.

## Materials and methods

### Plant material

Tomato (*Solanum lycopersicum* L. cv. Moneymaker) fruits of wild-type (WT) and independent *SlAREB1* transgenic lines (Orellana *et al.*, 2010) were grown in greenhouse conditions (22–24 ºC with a 16h light photoperiod). Individual flowers were labelled at anthesis in order to follow fruits through developmental stages. Fruits were harvested at the immature green (IG), mature green (MG), and red ripe (RR) fruit stages, and pericarp tissue was sampled as previously described (Bastías *et al.*, 2011).

### Metabolite analyses and total protein content determination

For metabolite analysis in pericarp tissue of WT and *SlAREB1* transgenic lines, the extraction, derivatization, and sample injection for GC-TOF-MS were performed according to Osorio *et al.* (2012). Identification and quantification of the compounds were performed with TagFinder 4.0 software and the mass spectra were cross-referenced with those in the Golm Metabolome Database (Kopka *et al.*, 2005; Schauer *et al.*, 2005). For secondary metabolite profiling, a non-targeted liquid chromatography-electrospray ionization-quadropole (LC-ESI-Qq)TOF-MS analysis was performed as described in Argamasilla *et al.* (2013) using a Waters Alliance HPLC system (Waters Corp., MA, USA) coupled to a QTOF I mass spectrometer (Micromass Ltd, Manchester, UK) through an electrospray source operated in positive mode. Mass chromatograms were centroided with Masslynx 4.1 software (Micromass Ltd) using Leu-enkephalin ([M+H]^+^ 556.2771) as lock-mass reference and converted to netCDF for subsequent XCMS processing. Mass chromatographic features accounting for individual metabolites were grouped using the CAMERA R package (Kuhl *et al.*, 2012) and only peaks with maximum area values within each group were used for principal component analysis (PCA). Metabolite identification and annotation were performed using the Metabolome Tomato Database (MoTo DB; Moco *et al.*, 2006). The total protein content was determined as described by Bradford (1976).

### Gene expression analysis by qRT-PCR

Three independent samples (biological replicates) of total RNA were obtained from pericarp tissue according to Bastías *et al*. (2011). Fruits were harvested, flash-frozen in liquid nitrogen, and kept at –80 ºC. Total RNA integrity was verified by agarose gel electrophoresis and their purity (*A*_260_/*A*_280_ >1.95) with a NanoDrop ND-1000 spectrophotometer (NanoDrop Technologies, Wilmington, DE, USA). cDNA synthesis and qRT-PCR was performed as described by Bastías *et al*. (2011) using a real-time PCR quantification system (MX3000; Stratagene, La Jolla, CA, USA) and the Brilliant II SYBR Green QPCR Master mix (Stratagene). Relative quantification of target expression genes was performed using the comparative 2(–Delta Delta) Ct method. The constitutively expressed elongation factor 1α of *S. lycopersicum* (*SlEF1α*, accession no. X14449) was used as reference gene for normalization of the expression. The primer pairs used for each gene are given in Supplementary Table S1 available at *JXB* online.

### Statistical analyses

Data mean comparisons were performed with SPSS 12.0 for Windows (SPSS Inc., Chicago, IL, USA). One-way analysis of variance (ANOVA) was performed to assess differences between control and transgenic fruits considering a significance value of 0.05. PCA was performed using the GINKGO multivariate analysis software (http://biodiver.bio.ub.es/vegana) and a heatmap representation was performed using the publicly available analysis software DChip (http://www.hsph.harvard.edu/cli/complab/dchip/)

## Results

### Overexpression of *SlAREB1* changes the metabolite composition of tomato fruits

**Fig. 1.**
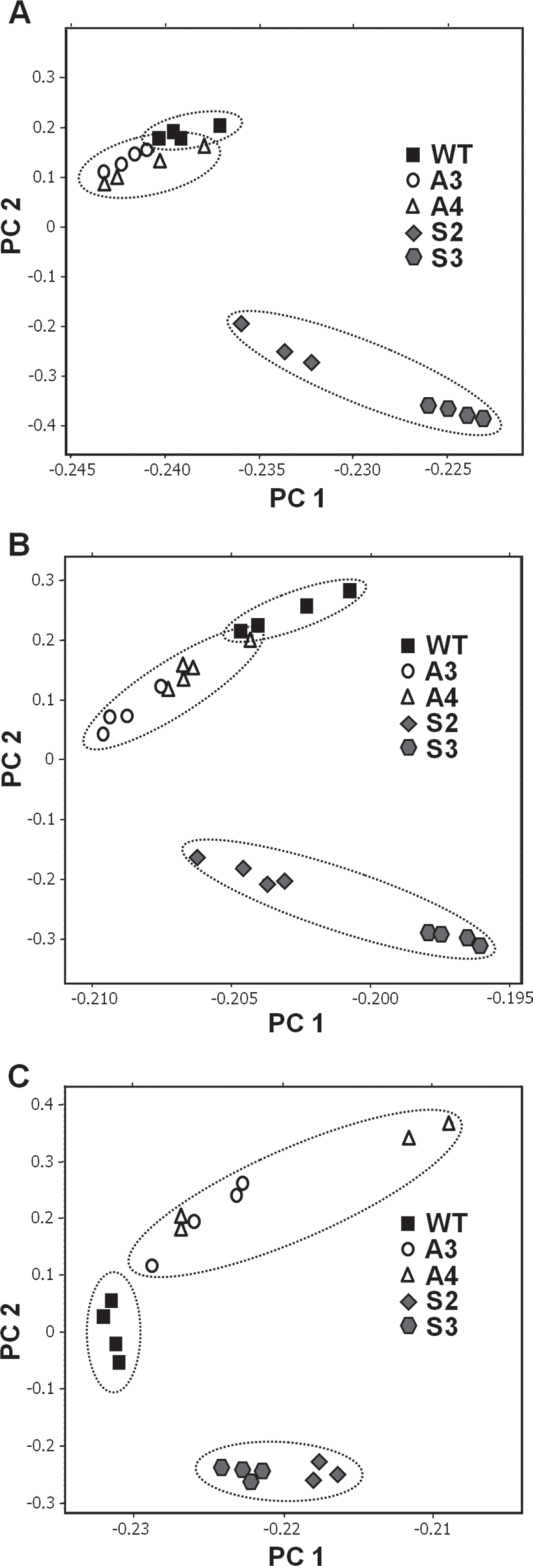
Differences between metabolic profiles of WT and *SlAREB1* transgenic fruit pericarp identified by principal component analysis (PCA) of GC-MS data. PCA of metabolic profiles obtained by GC-MS analysis of pericarp from (A) immature green, (B) mature green, and (C) red ripe fruits of WT and *SlAREB1*-overexpressing lines (S2 and S3) and *SlAREB1* antisense lines (A3 and A4). The percentage of the total variance explained by the two components is 98% in A, B, and C.

Transgenic *SlAREB1* tomato plants showed normal growth phenotype and the size and growth rate of their fruits were similar to those of WT plants (Bastías et al, 2011; Supplementary Fig. S1 at *JXB* online). Previous analysis of the main organic acids associated with organoleptic attributes of mature fruits suggested that expression of *SlAREB1* affects the concentrations of citrate, malate, and glutamate as well as those of fructose and glucose (Bastías *et al.*, 2011). To characterize further the role of this ABA-regulated transcription factor in the metabolic composition during fruit development, metabolite profiling by GC-TOF-MS and LC-MS was performed using fruit pericarp from both *SlAREB1*-overexpressing (OE) lines (S2 and S3) and antisense suppression (AS) lines (A3 and A4). GC-TOF-MS analysis allowed the detection of relative amounts of ~40 metabolites. The content of metabolites was measured in three stages of fruit development [immature green (IG), mature green (MG), and red ripe (RR)] in fruit flesh of the selected transgenic lines (Supplementary Table S2). A first examination of the differences in metabolite profiles between WT and transgenic fruits was attained with a PCA analysis of the GC-TOF-MS and LC-MS data sets. PCA of the GC-TOF-MS data could distinguish metabolite profiles of WT, OE, and AS lines. There was an evident difference between the OE lines and WT and AS plants at the IG and MG stages. At the RR stage, the difference was clear among the three groups of plants (Fig. 1).

**Fig. 2.**
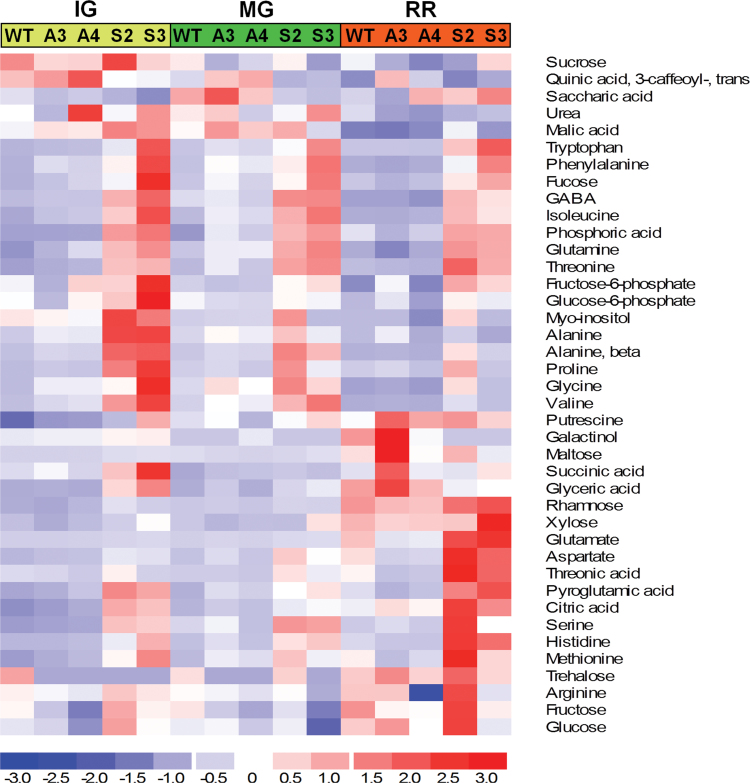
Metabolic profiles of tomato WT and *SlAREB1* transgenic fruit pericarp tissues. Heatmap of metabolite contents in fruits harvested at the immature green (IG), mature green (MG), and ripe red (RR) stage from wild-type (WT), antisense (A3 and A4), and overexpressing (S2 and S3) lines (four biological replicates). Colour changes represent the median of log 2 values obtained with the DChip software (http://www.hsph.harvard.edu/cli/complab/dchip/).

Results of the metabolic profile found by GC-TOF-MS analysis of WT and transgenic lines used this study are presented in Supplementary Tables S2–S5 at *JXB* online. In summary, at the IG, MG, and RR stages, ~34, 27, and 26 compounds were present at significantly higher abundances in the OE lines than in the WT, respectively. To simplify the interpretation of the metabolic changes, the relative metabolic content is presented in a heatmap (Fig. 2). Most metabolites identified are involved in primary carbon (C) and nitrogen (N) metabolism and exhibited a different pattern of accumulation between OE lines and WT or AS lines through the three developmental stages. At first sight, some metabolites stood out in all three stages of development whereas others were present in higher amounts only at the RR stage (Fig. 2). In the first group, amino acids such as tryptophan, phenylalanine, isoleucine, glutamine, threonine, alanine, proline, glycine, and valine, sugars such as fucose, fructose-6-phosphate, glucose-6-phosphate, and *myo*-inositol, γ-aminobutyrate (GABA), and phosphoric acid were notably higher in the OE lines than in the WT, especially in the first two developmental stages. Metabolites that were predominantly more abundant in the OE lines at the RR stage were the organic acids threonate, malate, and citrate and the amino acids glutamate, aspartate, pyroglutamate, serine, histidine, and methionine (Fig. 2; Supplementary Table S5). A few metabolites were detected at lower levels in the OE lines in comparison with the WT or AS lines: reduced amounts of quinate and saccharic acid were recorded at the IG and MG stages, and glycerate and galactinol were significantly decreased in OE lines at the RR stage (Fig. 2). Only in a few instances were there differences in the content of certain metabolites between the two overexpressing lines (e.g. sucrose) during the different developmental stages. Any difference in the rate of assimilate allocation could account for the minimal difference in fruit size observed in the S3 line (Supplementary Fig. S1 at *JXB* online). ANOVA *P*-values indicating the statistical significance of changes in metabolite contents related to the genotypes and developmental stages as well as the interaction between these two factors are supplied in Supplementary Table S6. This analysis additionally showed interactions between the developmental stage and genotype analysed. The results of these point-by-point comparisons are consistent with the PCA and indicate the differences in the primary metabolite profiles among the genotypes.

Changes in the numbers of putative secondary metabolites in OE and AS tomato fruits were determined by non-directed LC-MS. A relatively high number of compounds showed a significant difference in abundance between genotypes and developmental stages (Table 1). PCA was able to distinguish between the metabolic profiles of fruit pericarp from WT and OE or AS lines (Supplementary Fig. S2 at *JXB* online). Attempts to identify putative metabolites and their chemical formula using the tomato metabolite database MoToDB and literature surveys allowed the annotation of a few of them as flavonols and hydroxycinnamic acids (Supplementary Table S7). For example, in both OE IG fruits, the amount of some phenolics (feruloylquinic and chlorogenic acids) and the flavonone naringenin increased, whereas the content of chlorogenic acid and the flavonoids kaempferol-rutinoside, isoquercitrin, and rutin were reduced in the same fruits at the RR stage. Astragaline was the only identified flavonone to be more abundant in AS fruits compared with the WT (Suppementary Table S8).

**Table 1.** Differences in adducts between genotypes as determined by LC-MSSummary of the number of putative metabolites that showed significant differences in abundance (*P*≤0.01) between fruit pericarp from overexpression (OE) or antisense (AS) lines and wild-type plants (WT) during fruit development as determined by non-targeted LC-MS.

>Stage of development	>WT versus AS	>WT versus OE
Immature green	234	333
Mature green	68	112
Red ripe	105	332
Total	407	777

To determine whether accumulation of amino acids in the OE lines was due to an increase in the content of amino acids in the leaves that can be transported to the fruits, a GC-TOF-MS profiling analysis was performed in leaves of an OE line. Comparison of leaf and fruit data shows that out of the 29 compounds that accumulated more in the RR fruits of an OE line than in the WT, only three amino acids (arginine, proline, and threonine) also accumulated in the leaves of the OE line (Supplementary Table S9 at *JXB* online), suggesting that most changes in fruits were most probably due to an organ-specific activity of the transgene. Total protein content was also measured in the fruit pericarp to gain further insight into amino acid metabolism. Significant differences were only evident for the OE line S3 which displayed a higher total protein content in all three stages of development (Supplementary Fig. S3). These results suggest that the higher content of amino acids in the OE lines cannot be explained by either an increased amino acid biosynthesis in the vegetative tissue or an enhanced fruit protein degradation, and thus SlAREB1 probably functions at the transcriptional level to regulate the metabolism in the fruits.

### Expression of *SlAREB1* alters transcription of genes involved in primary metabolic pathways in tomato fruits

**Fig. 3.**
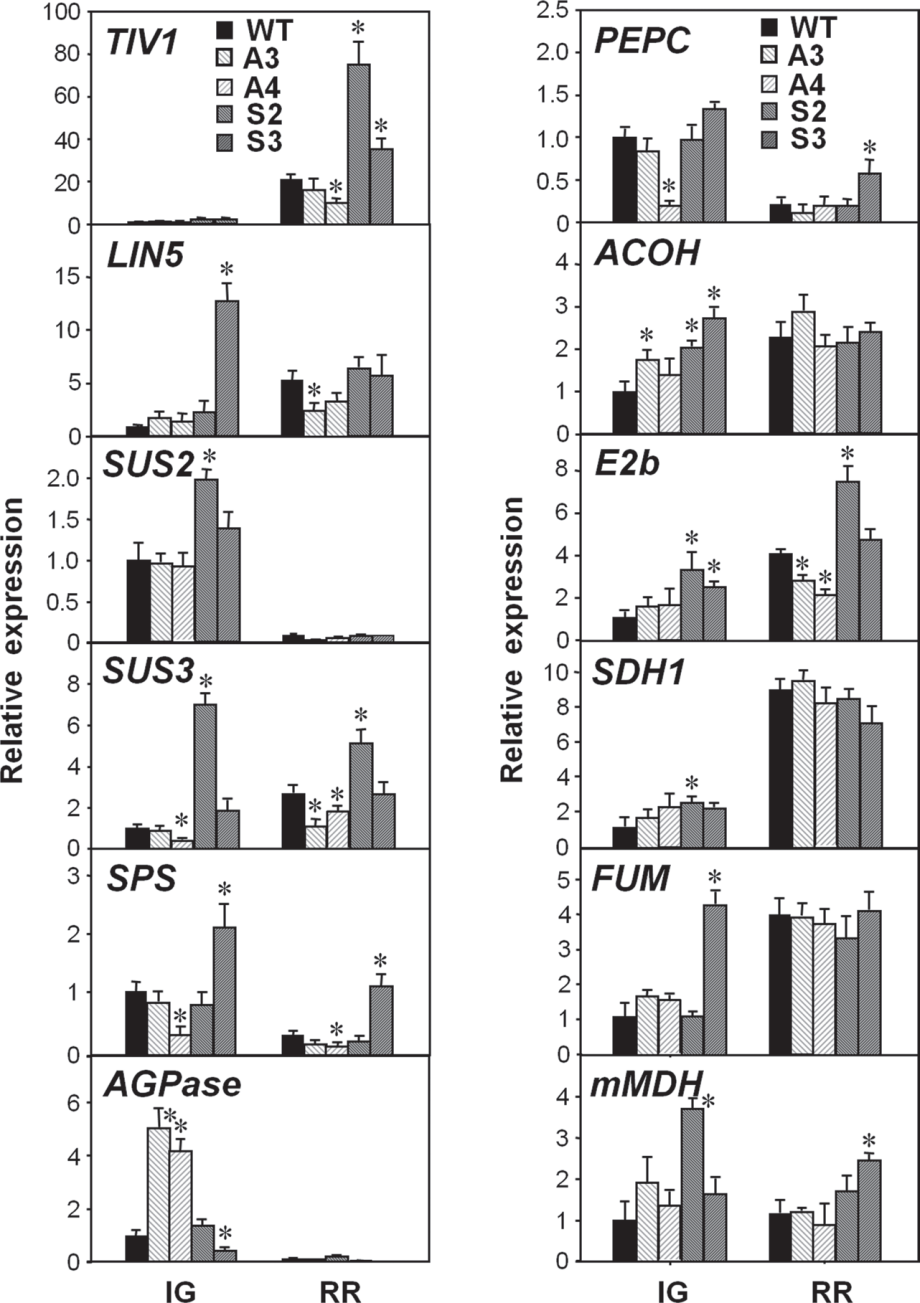
Expression of genes associated with sugar metabolism in WT and transgenic *SlAREB1* fruits. qRT-PCR analysis of genes encoding the tomato vacuolar invertase 1 (*TIV1*), cell wall invertase (*LIN5*), sucrose synthase 2 (*SUS2*), sucrose synthase 3 (*SUS3*), sucrose phosphate synthase (*SPS*), ADP-glucose pyrophosphorylase (*AGPase*), phosphoenolpyruvate carboxylase (*PEPC*), aconitate hydratase (*ACOH*), α-keto acid dehydrogenase, branched chain (*E2b*), succinate dehydrogenase 1 (*SDH1*), fumarase (*FUM*), and mitochondrial malate dehydrogenase (*mMDH*) in pericarp of immature green (IG) and ripe red (RR) fruits of wild-type (WT), AS (A3, A4, light hatched bars), and OE (S2, S3, dark hatched bars) lines. Bars indicate mean relative expression ±SE (*n*=3) normalized against *EF1α*. Asterisks indicate significant differences in transcript abundance compared with the WT (*P*≤0.05).

**Fig. 4.**
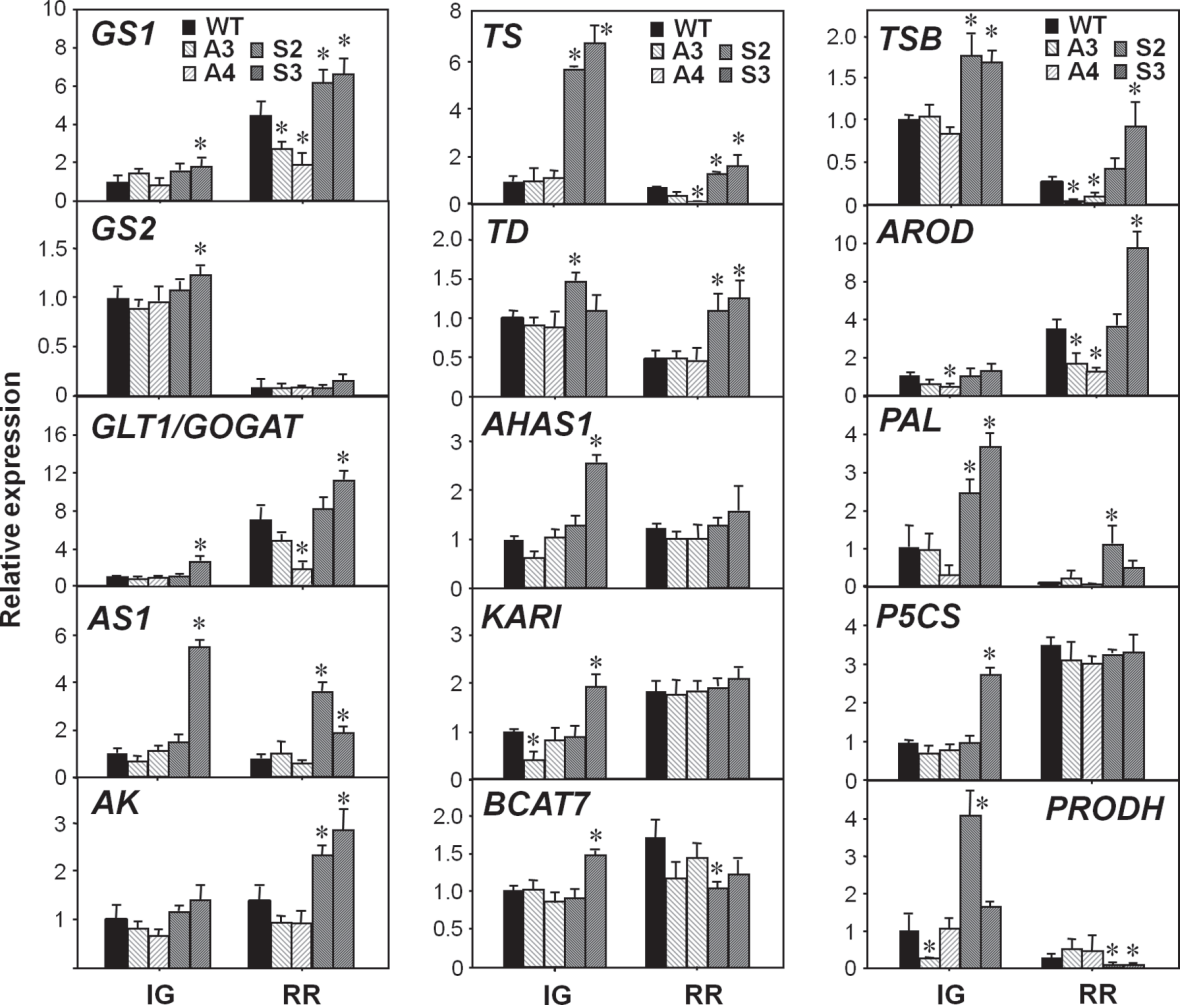
Expression of genes associated with amino acid metabolism and transport in WT and transgenic *SlAREB1* fruits. qRT-PCR analysis of the genes encoding glutamine synthetases (*GS1* and *GS2*), glutamate synthase (*GLT1/GOGAT*), asparagine synthetase (*AS1*), aspartate kinase (*AK*), threonine synthase (*TS*), threonine deaminase (*TD*), acetohydroxyacid synthase (*AHAS1*), ketol acid reductoisomerase (*KARI*), branched-chain aminotransferase 7 (*BCAT7*), tryptophan synthase b (*TSB*), arogetante dehydratase (*AROD*), phenylalanine ammonia lyase (*PAL*), delta-1-pyrroline-5-carboxylate synthetase (*P5CS*), pyrroline-5-carboxylate reductase (*P5CR*), and proline dehydrogenase (*PRODH*) in pericarp of immature green (IG) and ripe red (RR) fruits of wild-type (WT), AS (A3, A4, light hatched bars), and OE (S2, S3, dark hatched bars) lines. Bars indicate mean relative expression ±SE (*n*=3) normalized against *EF1α*. Asterisks indicate significant differences in transcript abundance compared with the WT (*P*≤0.05).

In view of the type of metabolites identified as exhibiting a different pattern of accumulation between WT and OE lines, special attention was focused on genes involved in pathways of primary C and N metabolism. Since a number of these genes are not present in tomato commercial microarrays, qRT-PCR analysis of genes encoding relevant enzymes of these metabolic pathways was performed at the IG and RR stages. Among the genes related to carbohydrate metabolism, those involved in sucrose metabolism were first analysed. They included genes for tomato vacuolar invertase 1 (*TIV1*), two cell wall invertases (*LIN5* and *LIN7*), sucrose synthase 2 and sucrose synthase 3 (*SUS2* and *SUS3*), and sucrose phosphate synthase (*SPS*) (Fig. 4; Supplementary Fig. S4 at *JXB* online). The expression of *LIN7* was similar among the genotypes within each developmental stage (Supplementary Fig. S4). In OE lines, increased expression of *LIN5*, *SUS2*, *SUS3*, and *SPS* was observed at the IG stage and of *TIV1*, *SUS3*, and *SPS* at the RR stage in at least one of the lines. Conversely, these genes were down-regulated in at least one AS line compared with the WT. In addition, expression of *AGPase* encoding the small subunit of ADP-glucose pyrophosphorylase involved in starch synthesis was found to be up-regulated in both AS lines and down-regulated in one OE line only in IG fruits (Fig. 3). In relation to enzymes of the tricarboxylic acid (TCA) cycle which controls the metabolic flow of the substrates for amino acid biosynthesis, the expression of *ACOH* (encoding aconitase), *E2b* (the branched chain of α-keto acid dehydrogenase), *SDH1* (succinate dehydrogenase 1), *FUM* (fumarase), and *mMDH* (malate dehydrogenase) was up-regulated in at least one OE line at the IG stage, whereas the transcript levels of *PEPC* (phosphoenolpyruvate carboxylase; that plays a role in replenishing the TCA cycle intermediates that are depleted in amino acid synthesis), *E2b*, and *mMDH* were significantly increased in one OE at the RR stage (Fig. 3).

Another group of genes analysed included key genes of different amino acid biosynthetic pathways. First, genes involved in the metabolism of glutamate and glutamine that share important routes in plants for the assimilation of reduced N in primary metabolism were evaluated. Among these, those encoding glutamine synthetase 1 (*GS1*), glutamate synthase (*GLT1/FdGOGAT*), and asparagine synthetase (*AS1*) were up-regulated in the OE lines and, in the case of *GS1* and *GLT1/GOGAT*, were also down-regulated in AS lines (Fig. 4). Genes involved in the synthesis of amino acids from oxaloacetate and pyruvate which comprise the aspartate family including the branched-chain amino acids were analysed. Genes for aspartate kinase (*AK*), threonine synthase (*TS*), and threonine deaminase (*TD*) were significantly up-regulated in the OE lines. In addition, acetohydroxyacid synthase (*AHAS1*), ketol acid reductoisomerase (*KARI*), and branched-chain aminotransferase 7 (*BCAT7*) were up-regulated in one OE line at the IG stage whereas *BCAT1* and *BCAT7* were down-regulated in the OE lines in RR fruits (Fig. 4). Genes implicated in the metabolism of proline and arginine were analysed. These amino acids are synthesized from 2-oxoglutarate and share routes with the metabolism of glutamate and polyamines. In this case, we analysed those for delta-1-pyrroline-5-carboxylate synthetase (*P5CS*), pyrroline-5-carboxylate reductase (*P5CR*), proline dehydrogenase (*PRODH*), *N*-acetyl-γ-glutamyl-phosphate reductase (*AGPR*), and arginase (*ARG2*) (Fig. 4; Supplementary Fig. S4 at *JXB* online). Of those, only *P5CS* was up-regulated in one OE line in green fruits, and *PRODH*, which is responsible for proline degradation, was also up-regulated in one OE line in green fruits but was down-regulated in OE RR fruits. Those controlling arginine synthesis (*AGPR*) and catabolism (*ARG2*) did not show significant differences between the genotypes (data not shown). Lastly, relevant genes for the synthesis of aromatic amino acids were investigated. The genes encoding tryptophan synthase b (*TSB*), arogenate dehydratase (*AROD*), and phenylalanine ammonia lyase (*PAL*) exhibited significant differences between WT, AS, and OE lines. The expression of these genes was higher in the OE lines at both stages of development, and in some cases the expression was down-regulated in the AS lines (Fig. 4). These results suggest that SlAREB1 could be affecting the amino acid content during the course of fruit development by regulating the expression of genes involved in their metabolism.

Due to the number of genes involved in amino acid metabolism that displayed differential expression in the transgenic lines compared with WT plants, the 5’ upstream regions of >50 genes associated with amino acid metabolic pathways were scrutinized for the presence of ABRE *cis*-elements which are known to be recognized by AREB transcription factors. Genomic sequence information for the genes was obtained from the SGN database (http://solgenomics.net) and the promoter analysis performed using the cis-acting regulatory element database PlantCare (http://bioinformatics.psb.ugent.be/webtools/plantcare/html/). All genes up-regulated in the OE lines in any of the stages analysed presented at least one putative ABRE within 1kb of their respective upstream regulatory region (Supplementary Table S10 at *JXB* online).

### Ripening-associated genes are also altered in *SlAREB1* transgenic lines

**Fig. 5.**
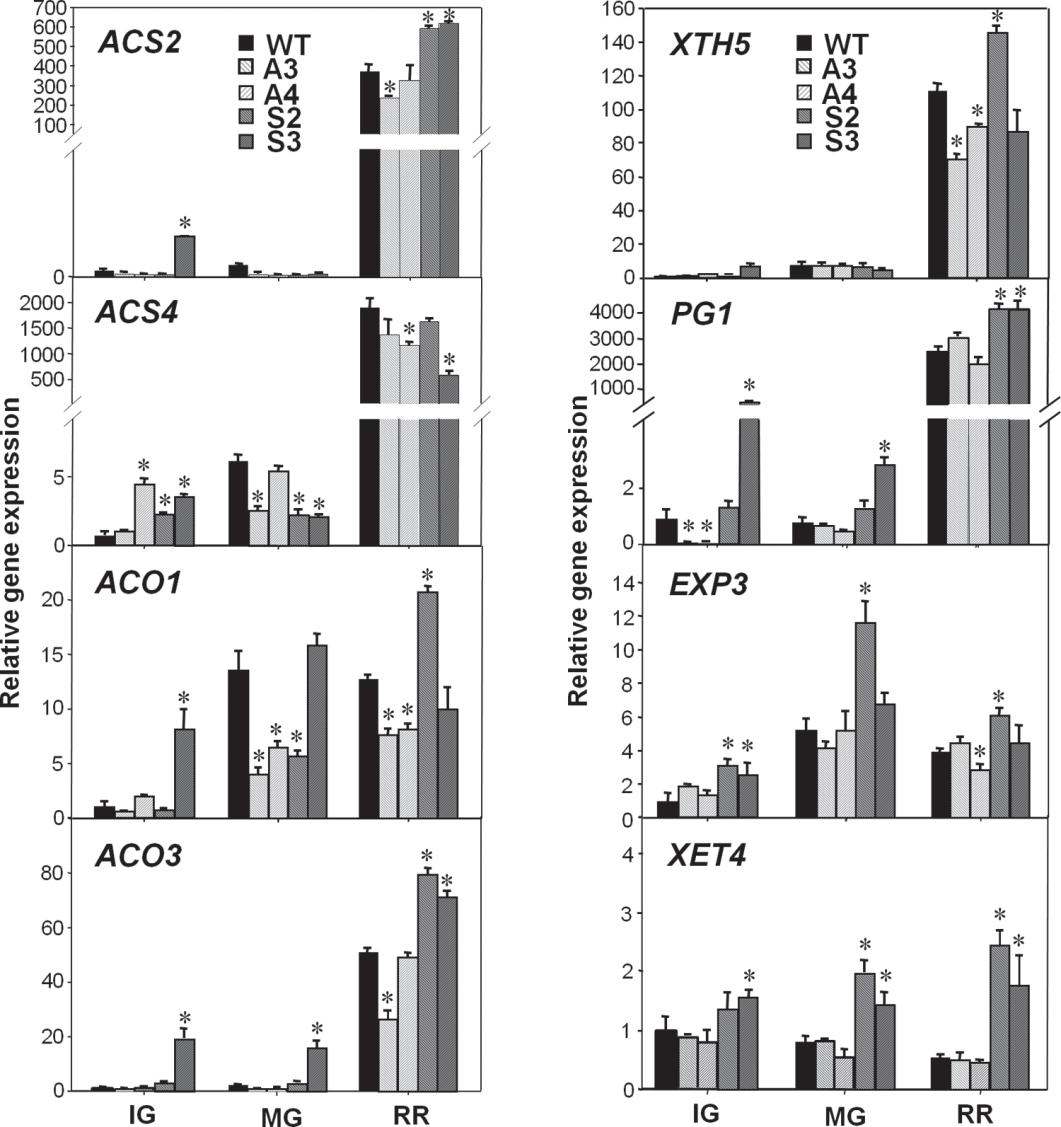
Expression of genes associated with fruit ripening in WT and transgenic *SlAREB1* fruits. qRT-PCR analysis of genes related to ethylene synthesis: ACC synthase 2 (*ACS2*), *ACC synthase 4* (*ACS4*), ACC oxidase 1 (*ACO1*), and ACC oxidase 3 (*ACO3*); and to cell wall catabolism: xyloglucan endotransglycosylase/hydrolase 5 (*XTH5*), polygalacturonase 1 (*PG1*), expansin 3 (EXP3), and xyloglucan endotransglycosylase 4 (*XET4*) in pericarp of immature green (IG), mature green (MG), and ripe red (RR) fruits of wild-type (WT), AS (A3, A4, light hatched bars), and OE (S2, S3, dark hatched bars) lines. Bars indicate mean relative expression ±SE (*n*=3) normalized against *EF1α*. Asterisks indicate significant differences in transcript abundance compared with the WT (*P*≤0.05).

It has been described that exogenous ABA can induce the expression of some genes associated with ethylene biosynthesis (Zhang *et al.*, 2009). To elucidate whether SlAREB1 has an effect on genes involved in ethylene production, the relative gene expression of *SlACS2*, *SlACS4*, *SlACO1*, and *SlACO3* was analysed in the transgenic fruits at three stages of development. Whereas there was no correspondence between the expression levels of *SlACS4* and that of the transgene, expression of *SlACS2* at the RR stage was higher in both OE lines compared with the WT (Fig. 5). In addition, transcript levels of *ACO1* and *ACO3* were also increased in at least one OE line in all three stages and *ACO1* was lower in the two AS lines compared with the WT at MG and RR stages (Fig. 5). Expression of other genes associated with cell wall modification relevant to fruit ripening was also analysed. They included genes for xyloglucan endotransglucosylase-hydrolase 5 (*XTH5*), polygalacturonase 1 (*PG1*), expansin 3 (*EXP3*), and xyloglucan endotransglycosylase 4 (*XET4*). Both AS lines exhibited lower expression of *XTH5* than the WT at the RR stage, and one OE line showed a higher expression of the gene compared with the WT. The difference in expression of *PG1*, *EXP3*, and *XET4* between OE lines and the WT was also evident from the immature stage through ripening (Fig. 5). Although these differences in gene expression did not represent significant changes in fruit softening during the ripening stage between the genotypes, the average fruit firmness tended to be lower in OE lines compared with WT and AS lines (Supplementary Fig. S5 at *JXB* online).

### Metabolic programming in maturing fruits is affected in *SlAREB1* OE lines

**Fig. 6.**
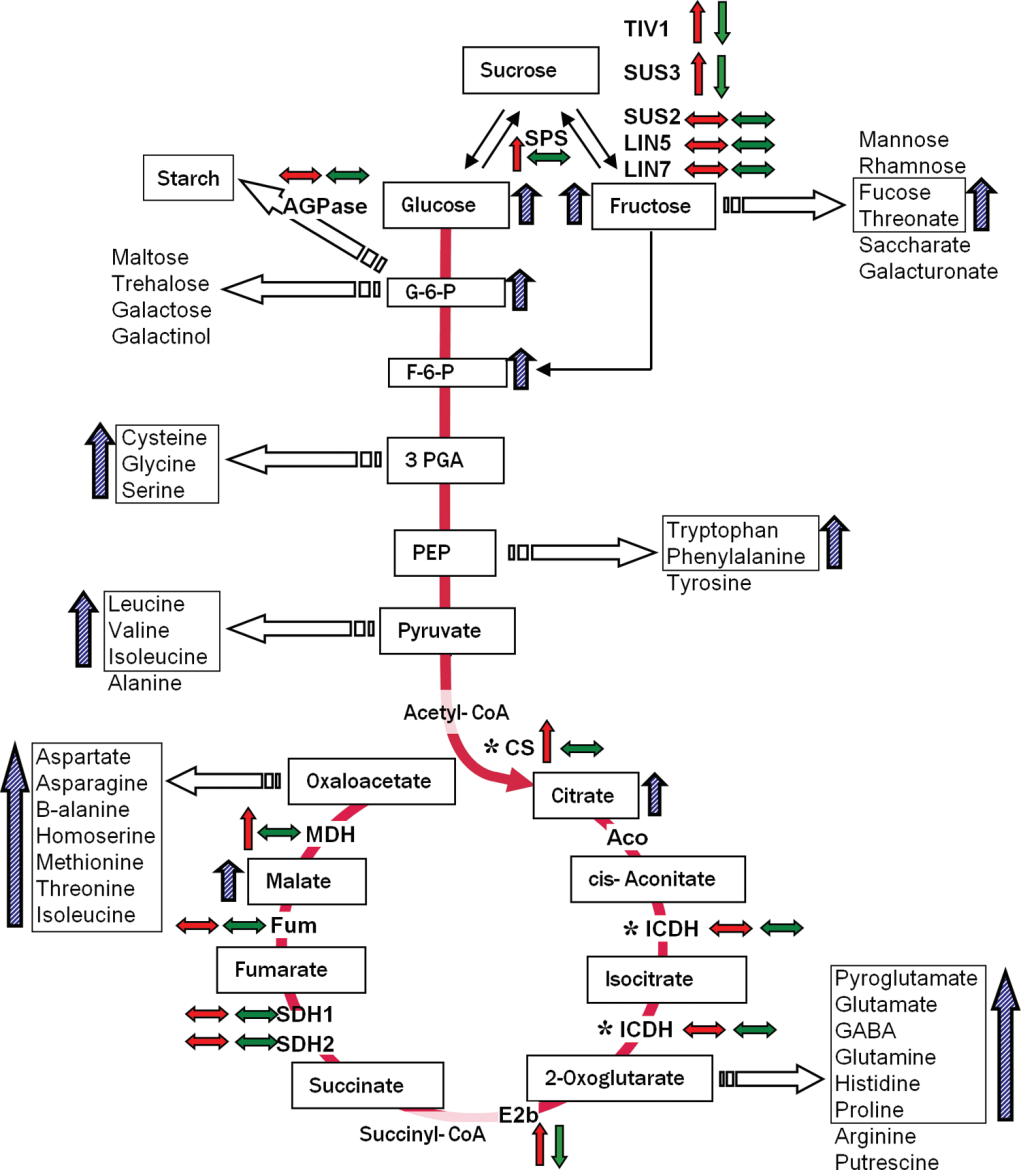
Modulation of primary metabolism by SlAREB1 in red ripe fruits. Summary of changes in gene expression and metabolite contents in *SlAREB1* transgenic red ripe fruits compared with the WT. Small paired arrows indicate the gene expression in OE (red left arrow) and AS (green right arrow) lines, respectively, compared with the WT (e.g. horizontal double-headed arrows indicate similar expression to the WT). Hatched blue arrows refer to increases in metabolite content (in boxes) in OE fruits compared with the WT. Asterisks indicate genes described in Bastías *et al*. (2011). (This figure is available in colour at *JXB* online.)

A summary of the metabolite content and gene expression results found in tomato fruits at the RR stage is shown in Fig. 6. The diagram summarizes the main pathways of carbohydrate metabolism including glycolysis and the TCA cycle. This metabolic backbone generates sugars and organic acids, while some intermediates operate as links to amino acid biosynthesis. Compared with the WT, *SlAREB1* transgenic RR fruits accumulated more fructose and glucose which can be associated with the higher expression of *TIV1* and *SUS3*. Also OE lines displayed a higher content of sugar phosphates and some organic acids such as citrate and malate. However, the most striking differences correspond to the greater accumulation of amino acids from all groups (the pyruvate, aspartate, glutamate, and aromatic families) in fruits of OE lines compared with those of WT plants. In general, the higher amino acid content observed correlates with the expression trend of some genes implicated in the metabolism of amino acids (Fig. 4).

## Discussion

Increasing evidence indicates that hormones, including ABA, control different phenotypic characteristics of fruits from the setting process to ripening (Srivastava and Handa, 2005; Vriezen *et al.*, 2008; Wang *et al.*, 2009; Zhang *et al.*, 2009; Nitsch *et al.*, 2012; Sun *et al*., 2012*a*,*b*). Nonetheless, the molecular mechanisms by which ABA participates in these processes are not sufficiently clear. This work describes a possible new function for an ABA-regulated transcription factor, SlAREB1, in controlling primary biochemical pathways and metabolic attributes of the tomato fruit during development and ripening.

### Effects on the metabolic profile

A global metabolic profile analysis by GC-TOF-MS identified several metabolites that are clearly affected when the expression of *SlAREB1* is modified. A factorial ANOVA shows that a large proportion of metabolites was affected by the genotype or developmental stage, or presented an interaction between them (Supplementary Table S6 at *JXB* online). Among all metabolites, fruit pericarp of OE lines accumulated notably more of most amino acids than WT or AS lines (Fig. 2; Supplementary Tables S3–S5). It is well known that the content of free amino acids, especially tryptophan, aspartate, and glutamate, increases in tomato fruit pericarp during ripening (Carrari *et al.*, 2006) as observed also in this study (Supplementary Table S2). In growing fruits, accumulation of amino acids is generally due to the transport of resources to the sink tissue. A comparison of the metabolite contents between WT and OE leaves and fruits on plants at the flowering stage revealed different significantly altered metabolites of which only a few overlapped between the two organs. Even though this measurement depicts a metabolic condition at a given moment during development, the data suggest that this difference is a consequence of overexpressing the transcription factor. This assumption is supported by the observed effect of altering the expression of *SlAREB1* on the response of several genes controlling the biosynthesis of amino acids in the pericarp tissue. Interestingly, such an effect caused a change in at least 15 and 10 nitrogenous compounds in mature red fruits of OE and AS lines, respectively (Supplementary Table S5). In addition, it has been suggested that the increment in amino acid concentration also may be due to peptidase activities in the fruit pericarp (Sorrequieta *et al.*, 2010). Total protein content usually does not diminish in WT pericarp; however, a rapid protein turnover which is much more difficult to determine should also take place if this degradation is produced. Considering that protein content in OE lines did not diminish compared with the WT (Supplementary Fig. S3), the data suggest that the increment in amino acids in OE lines is not a result of total protein degradation but most probably of *de novo* synthesis.

Overexpression of *SlAREB1* also had an effect on C metabolism. At the RR stage, the amounts of fucose, fructose-6-phosphate, glucose-6-phosphate, and the TCA cycle acids citrate and malate were clearly increased in the OE lines. On the other hand, variations in the content of sucrose, glucose, and fructose were not significant among the genotypes. The implication of organic acids for the organoleptic properties of fruits is well established; however, the metabolism dynamics related to the TCA cycle in fruits are not totally understood. This cycle connects glycolysis with the organic acid and amino acid biosynthetic pathways performing important roles in the generation of ATP and NADH (Yin *et al.*, 2010). Different interactions exist between the TCA cycle and GABA metabolism. GABA is one of the most abundant nitrogenous compounds in tomato fruits; however, its physiological role is not clear. The maximum accumulation of GABA occurs at the IG stage, representing ~50% of the total amino acid content, and then decreases during ripening (Akihiro *et al.*, 2008). A new pathway connecting GABA metabolism to the C/N supply during tomato fruit development has been described. Before ripening, GABA is converted to malate via succinate semialdehyde (Bouché and Fromm, 2004) or can also be diverted through pyruvate to re-enter the TCA cycle flux and be saved as citrate and contribute to respiration during fruit ripening (Yin *et al.*, 2010). Stimulation of GABA synthesis can also be regulated by ABA and abiotic stress (Bouché and Fromm, 2004). GABA content in fruit pericarp increased in all stages of *SlAREB1*-overexpressing fruits, suggesting that this transcription factor may be controlling its metabolism.

The accumulation of organic acids and amino acids that can affect the organoleptic properties of fruits observed in OE lines is comparable with that described in tomato fruits from plants grown under salt, drought, high light, and high temperature stresses (Balibrea *et al.*, 2000; Rosales *et al.*, 2007; Urano *et al.*, 2009; Yin *et al*., 2009, 2010). This accumulation can be the result of the activation of C and N metabolism (Yin *et al.*, 2010). However, the molecular mechanisms controlling such metabolic changes in fruits under stress conditions are still puzzling. Some of these metabolites have a role as compatible osmolytes, and their build up in plants occurs as an acclimation response to stress conditions. In particular, the accumulation of branched-chain amino acids (BCAAs) is induced many-fold during osmotic stress. However, with the exception of proline, not much research has been focused on understanding the function of BCAAs and other amino acids (Joshi *et al.*, 2010). That said, they have been noted to accumulate following drought stress in a range of species (Obata *et al.*, 2012; Witt *et al.*, 2012). Although the increment of certain assimilates under stress conditions is not fully understood, some of them could participate as metabolic precursors of antioxidant compounds that restrict the accumulation of reactive oxygen species, reducing the incidence of cell necrosis and subsequent production losses (Rosales *et al.*, 2007; Yin *et al.*, 2010). A control mechanism involving oxidative stress and hormones may take place in fruits. In this sense, vitamin C content in tomato lines correlated with the expression of genes involved in hormone signalling (i.e. ethylene, ABA, gibberellins, and auxin) rather than with genes involved in ascorbate synthesis and, in turn, the level of gene expression is affected by the oxidative status of the fruit (Lima-Silva *et al.*, 2012).

Secondary metabolites are important during ripening because some are responsible for the main flavour and aroma characteristics of RR tomato. The PCA analysis of the LC-MS profiles of fruit pericarp showed significant differences between the transgenic lines and the WT (Supplementary Fig. S2 at *JXB* online). ABA has been described to regulate the degree of pigmentation and carotenoid composition during tomato ripening (Sun *et al.*, 2012*b*). High accumulation of secondary metabolites such as carotenoids and anthocyanins has also been detected in tomatoes grown under salinity stress (Borghesi *et al.*, 2011). Expression of *PAL*, involved in secondary metabolism, was higher in OE lines than in WT and AS lines at the IG stage (Fig. 5). *SlAREB1* OE lines could have an abiotic stress signal activated that would produce an increment in the content of some secondary metabolites such as flavonols and hydroxycinnamic acid derivatives (Supplementary Table S7). A targeted approach will be necessary to identify other secondary compounds affected by altering the expression of *SlAREB1*.

### Effect on gene expression

The expression of *SUS2* and *SUS3* was affected in transgenic fruits compared with the WT (Fig. 3). Both SUS and invertase activities participate in providing hexoses from translocated sucrose to developing fruits, despite the fact that it is thought that the main activity on sucrose hydrolysis is produced by soluble acid invertases (Nguyen-Quoc and Foyer, 2001). It has been shown that ABA can induce expression of acid invertase-encoding genes in fruits and regulate their enzymatic activity (Proels *et al.*, 2003). Transcript levels of the gene for the vacuolar invertase TIV1 are normally low during fruit growth and increase during the ripening process (Miron *et al.*, 2002). The expression of *TIV1* and *LIN5*, a cell wall-associated invertase expressed in fruits, was affected in *SlAREB1* transgenic lines. Expression and activity of TIV1 usually determine the level of sucrose accumulation in tomato fruits (Miron *et al.*, 2002) and its up-regulation most probably was responsible for the sucrose and hexose contents in the transgenic lines. The activity of TIV1 probably also superseded that of *SPS* in one OE line (Fig. 3). Although contents of glucose and fructose were not noticeably higher in the OE lines than in WT fruits, OE lines accumulated more glucose-6-phosphate and fructose-6-phosphate (Supplementary Table S5). This observation suggests that C metabolism is probably driven towards the TCA cycle and the synthesis of compounds derived from it.

Whereas the synthesis and degradation of citrate are known to be catalysed only by enzymes involved in the citric acid cycle, the levels of malate can be regulated by synthesis, transport, and enzymes not directly involved in its metabolism (Sweetman *et al.*, 2009). Citrate is accumulated mainly by the action of citrate synthase (CS). The expression the mitochondrial citrate synthase gene (*mCS*) was up-regulated in the pericarp of mature fruits in *SlAREB1* OE lines and less expressed in AS lines (Bastías *et al*, 2011). Expression of genes encoding aconitate hydratase (*ACOH*), the branched chain of α-keto acid dehydrogenase (*E2b*), succinate dehydrogenase 1 (*SDH1*), fumarase (*FUM*), and mitochondrial malate dehydrogenase (*mMDH*) was also up-regulated in *SlAREB1* OE immature fruits. In mature OE ripe fruits, the transcript levels of PEPC, E2b, and mMDH were also up-regulated (Fig. 3).

Among the genes associated with amino acid metabolism, statistical differences were found for *GS1*, *GLT1*, *AS1*, *AK*, *TS*, *TD*, *TSB*, *AROD*, and *PAL* between fruits of *SlAREB1* OE lines and the WT in at least one developmental stage (Fig. 5). The regulatory regions of all these genes contain one or more ABRE boxes (Supplementary Table S10 at *JXB* online) which are direct targets of bZIP factors such as AREBs, suggesting that *SlAREB1* could modulate gene expression changes associated with ripening alone or in combination with other transcriptional regulators. This regulation could be carried out directly by interaction with promoters or indirectly through a transcriptional cascade (Yoshida *et al.*, 2006). Nonetheless, not all metabolic pathways are controlled at the transcriptional level. It is also possible that the pathways altered in the fruits of *SlAREB1* transgenic lines may be regulated at the post-transcriptional level such as through RNA processing (Hu *et al.*, 2005), by traslational or post-traslational regulation (Kim *et al.*, 2003), or by feedback or epigenetic mechanisms (Luo *et al.*, 2012). The fact that the content of most amino acids was affected in the transgenic lines but the expression of only a few genes was modified could be explained by the fact that synthesis of several amino acids shares common precursors and pathways. In this respect, direct metabolic engineering strategies aiming to increase the content of one or more target amino acids have had an unanticipated effect on other amino acids (Joshi *et al.*, 2010).

### Effect on ripening

Mizrahi *et al.* (1975) described that application of ABA accelerated the ripening of detached tomato fruits. This response seemed to be independent of ethylene since ABA did not alter either respiration or ethylene production in WT fruits or induce ripening in the *rin* ripening mutant. *RIN* encodes a MADS-box transcription factor that acts as a key regulator of the onset of ripening by acting upstream of ethylene-mediated and non-mediated signalling (Martel *et al.*, 2011). However, it has been shown that the peak of ABA content precedes ethylene production in seeds and flesh and that exogenous ABA treatment induces the expression of both ACS and ACO genes, thus promoting ethylene biosynthesis (Zhang *et al.*, 2009). Additionally, it has been described that ABA can promote fruit ripening through the modulation of cell wall composition and ethylene-related gene expression in a developmental stage-dependent manner (Soto *et al.*, 2013). In this study, expression of genes involved in ethylene biosynthesis and cell wall modification was statistically different in *SlAREB1* transgenic lines compared with the WT (Fig. 5). These changes in the expression of ripening-related genes could be linked to the role of ABA described by Zhang *et al.* (2009). It has been reported that down-regulation of ABA synthesis and accumulation reduces the expression of genes encoding cell wall catabolic enzymes such as PG, XET, and pectin methyl esterase, and delays ripening of tomato fruits (Sun *et al.*, 2012*a*). Others have also suggested that ABA can control ethylene synthesis and regulate cell wall metabolism- and auxin-related genes (Sun *et al.*, 2012*b*; Soto *et al.*, 2013). This study suggests that transcription factors such as SlAREB1 can mediate the ABA signalling that regulates ripening through induction of ethylene biosynthetic genes and modification of cell wall metabolism.

Overexpression of *SlAREB1* could cause the activation of stress-related signalling in fruits that could, in turn, result in increased content of some metabolites. Although participation of other components of the ABA signalling pathway cannot be discarded, this work points to a direct or indirect role for SlAREB1 in the synthesis and modulation of compounds that bestow the biochemical, organoleptic, and nutritional properties of tomato fruits during their development and ripening. Although coordinated changes in the expression of regulatory genes such as transcription factors and the metabolic composition of tomato fruit have been demonstrated (Rohrmann *et al.*, 2011), such network analysis has also revealed a complex regulation of both primary and secondary metabolism. In this regard, the present study provides a new notion regarding the function of an ABA-regulated transcription factor and contributes to our understanding of the molecular processes that take place during fruit development and ripening.

## Supplementary data

Supplementary data are available at *JXB* online.

Figure S1. Characteristics of *SlAREB1* transgenic fruits.

Figure S2. Differences between metabolic profiles of wild-type and *SlAREB1* transgenic fruit pericarp identified by principal component analysis (PCA) of LC-MS data.

Figure S3. Total protein content in pericarp of *SlAREB1* transgenic tomato fruits.

Figure S4. Expression of genes associated with sugar and amino acid metabolism in wild-type and transgenic *SlAREB1* fruits.

Figure S5. Firmness of *SlAREB1* transgenic tomato fruits.

Table S1. Primer sequences used in real-time quantitative PCR analysis.

Table S2. Relative metabolite contents in fruit pericarp of wild-type tomato.

Table S3. Relative metabolite contents in pericarp of immature green fruits of *SlAREB1* transgenic lines.

Table S4. Relative metabolite contents in mature green fruit pericarp of Table S5. Relative metabolite contents in ripe red fruit pericarp of *SlAREB1* transgenic lines.

Table S6. Comparison of metabolite content between genotypes and developmental stages using a two-way analysis of variance.

Table S7. Identification of statistically different secondary metabolites in SlAREB1 transgenic tomato fruit pericarp with respect to wild-type fruit pericarp.

Table S8. Relative content of identified secondary metabolites in fruits of *SlAREB1* transgenic lines.

Table S9. Changes in analyte content in red ripe fruits and leaves in the S2 overexpressor line compared with the wild type.

Table S10. Presence of ABA response elements (ABREs) in the promoters of tomato genes associated with carbon and amino acid metabolism.

Supplementary Data

## Abbreviations:

ABA: abscisic acid
AREB: ABA-response element-binding factor
bZIP: basic leucine zipper
PCA: principal component analysis
qRT-PCR: quantitative reverse transcriptase–PCR
WT: wild type.
