# Supplementary material for: The transcription factor AREB1 regulates primary metabolic pathways in tomato fruits
Source: J Exp Bot. 2014 Mar 22;65(9):2351–63. doi: 10.1093/jxb/eru114 (PMC4036503; doi:10.1093/jxb/eru114)
Supplement: Supplementary Data [file supp_65_9_2351__index.html]

The transcription factor AREB1 regulates primary metabolic pathways in tomato fruits — The transcription factor AREB1 regulates primary metabolic pathways in tomato fruits — Supplementary Data 

# The transcription factor AREB1 regulates primary metabolic pathways in tomato fruits

## Supplementary Data

Data files

**Files in this Data Supplement:**

- Supplementary Data - Supplementary Data
- Supplementary Data - Supplementary Data
