# Supplementary material for: The transcription factor AREB1 regulates primary metabolic pathways in tomato fruits
Source: J Exp Bot. 2014 Mar 22;65(9):2351–63. doi: 10.1093/jxb/eru114 (PMC4036503; doi:10.1093/jxb/eru114)
Supplement: Supplementary Data [file supp_eru114_jexbot112896_file001.pdf]

The transcription factor SlAREB1 regulates primary metabolic pathways in tomato fruits
Bastías A, Yañez M, Osorio S, Arbona V, Gómez-Cadenas A, Fernie AR, Casaretto JA

Table S1. Primer sequences used in real time quantitative PCR analysis

Gene name	Accession	Primer	Forward primer (5' - 3')
EF1α	X14449	FWD	ACCACGAAGCTCTCCAGGAG
		REV	CATTGAACCCAACATTGTCACC
TIV1	M81081	FWD	AAGTGGACAAAGTCGCGCTT
		REV	TGGTCCCAAAATGCCTCTG
LIN5	AJ272304	FWD	TTGGAAGGGATTGAGAATCGA
		REV	CGGATTCATTTGACCAACCC
LIN7	AF506006	FWD	TTTGGTGCTGGTGGAAAGACA
		REV	GGCTCCGTTCCGTTGTTAAAC
SUS2	AJ011535	FWD	TTTGGTCTGACTGTTGTCGAAG
		REV	GTCGAAATGGTTTCCCAATG
SUS3	AJ011319	FWD	GAACGAAGAACACCTGTGTGT
		REV	TCTCTGCCTGCTCTTCCAA
SPS	AY726439	FWD	GATAGTGCTGCTCTCCTTTCAGG
		REV	TGGTGCTTGTGATAACGAGTTCTG
AGPase	L41126	FWD	TAAATCGCCACCTTTCACGG
		REV	CGGACTTTGTTGAGCAGCAAG
PEPC	SGN-U576244	FWD	GCACATCCCACGCAAATAAACC
		REV	AAACCAGCCCTAGCTTCATCAAC
ACOH	AY250115	FWD	TGCGATGAATCCTCTCGAAGCTC
		REV	TGCCAAGGGACCAATGTTGTTGG
OGDH-E2b	SGN-U568072	FWD	TTCAAACGGTGGAGTGTATGGAAG
		REV	TGCATTCCCAAGATAGCAGACTGG
SDH2-1	SGN-U563725	FWD	CTTTGGGCAAGCACGAGAACAG
		REV	CGCCTGGACGCAGTTAATGATG
SDH2-2	SGN-U563726	FWD	TTCGGACTCCCAAGCACAGAAC
		REV	TCTGGAGTCCATCGGTACATTCG
FUM	AK325138	FWD	TGCTCGTCACATGCTTGAACCC
		REV	CCTTGTGCGCTTTCTTGGCAAC
mMDH	SGN-U565568	FWD	ACGCTTGGTAAGTGCCTGAATGTC
		REV	TTTCCCAAGCCACTCCAAGTGC
GS1	AF200360	FWD	CATTGTTGACGCCCATTACA
		REV	ATGCCAACAGAAGGTCCAAC
GS2	U15059	FWD	CCCTTGAGGCTGAAGCTCT
		REV	GATGTTCAAAGTAGTCCAA
GLT1	SGN-U575484	FWD	CAG CCT TGA AGT TGG ACC TGT TG
		REV	TGT GAA AGG ACT GGA GGT GAT ACG
GDH	SGN-U276420	FWD	CCA GAG CAC AGG GTA TAA GAA CAT C
		REV	ATT CAC GAG ATA GGT GGC AAA GTA G
AST1	SGN-U567172	FWD	GGAGTAGAGGTTGATGGATTTGG
		REV	GGGACAGGAGGTCTGAGAGTAAC
AS1	AY240926	FWD	GCTCGATACTTGGCTGGAAC
		REV	TCGCAGCCTTAAGATCTGGT

AGPR	SGN-U562906	FWD	CTGGCCAATCAATCGAATCT
		REV	CAGCATCCACAGCAGAGAAA
AK	SGN-U569440	FWD	ATCAGTAGCCTCAGCCGAAA
		REV	GCCAGCAAAAGGTTGTTTGT
TS	SGN-U573081	FWD	CAGCCACAAACGATGCGATTTC
		REV	TGCCCTTCCAACCTCAAACTATTC
TD	BG628643	FWD	CAGGGTTAGGTTCAGGCAAA
		REV	TTCAAAGAGCCCACAAGTCC
AHASS1,2	SGN-U572742	FWD	CACTCCTGGGCCATACTTGT
		REV	TAGGAACGTCTCCCATCACC
KARI	SGN-U572725	FWD	GTGAGATCAGGAGCGTTGTCTTG
		REV	GCAGGTAAGCCCTCCTTTTCAT
BCAT1	SGN-U569626	FWD	AGGGCTCTATTTACTTCTTTTGAG
		REV	CATACACATTCTTTTAGCACCAATT
BCAT7	SGN-U565681	FWD	GTCATGGAGCTTGTGCTGAA
		REV	AGCTCCCCCATAGTTCCTGT
IGPS	SGN-U563660	FWD	GGA AGG AGA GCG TGG GGA AAG
		REV	TGG ATA GGA ACA AGT AGG AGT CAG C
TSB	SGN-U581112	FWD	TCA CAG ATG AGG AGG CTT TGG AAG
		REV	ACT CTT TGG GAC AAG TGT TAC GAA C
AROD	AI773917	FWD	GGCTTTGGAAACTTTGGACA
		REV	GGGATTGTGCTATGAGGGAAT
PAL	SGN-U232238	FWD	TCTTGTCCCGTTGTCATACATTGC
		REV	ACCATTGATGCCATACCAGAACC
P5CS	U60267	FWD	CAGTGGCTTTGCGACAGATA
		REV	CACGAGCATCAGTCTCTCCA
P5CR	SGN-U567857	FWD	AGGGGTGGCTGCTGGTTTAC
		REV	AACAACTGCATTCATCAAGATACCG
PRODH	SGN-U578070	FWD	CAGGAAAACTTGCTGCATCC
		REV	TGAAAGCCCTTCTGCCATAC
ACS2	AY326958	FWD	TGGAGAAAACAAGAGGAGGAAGA
		REV	GGCACCACCAGCCATAACA
ACS4	M63490	FWD	CCATCTTGTTTGCGACGAAATA
		REV	CGATGCTAACGAATTTTGGAGAA
ACO1	EF501822	FWD	AAATCATGAAGGAGTTTGCTGATAAA
		REV	TTTTCACACAGCAAATCCAACAG
ACO3	X58885	FWD	ACGGGAAGTACAAGAGCGTGAT
		REV	CTAGTGACATCCGAGTCCCATCT
XTH5	AY497475	FWD	GGATTCAGCCATCTCTTTGGTG
		REV	GAACCCTGAACCTGTGTTTTGG
PG1	X14074	FWD	GGCAATGGACAAGTATGGTGG
		REV	CAGAAGGTTAAGGCCGTTGGT
EXP3	AF059487	FWD	TTTGCGGAGCTTGCTTTGAA
		REV	GGAGCACAAAAATTCGTTGCAG
XET4	AF186777	FWD	CTGCTGGTGTTGTCACTGCT
		REV	ATGTATGGCTGACCGGTTCT

Table S2. Relative metabolite contents in fruit pericarp of wild type tomato. Metabolites were determined as described in Materials and Methods. Data are normalized with respect to mean response calculated for the wild type. Values are presented as means ± SE of four individual fruits per line, and values in boldface were determined by t test to be significantly different ($p \leq 0.05$) from immature green development stage. nd, not detected.

Development Stage	IG	MG	RR
**Amino acids**			
Alanine	1.000 ± 0.033	1.176 ± 0.097	0.785 ± 0.130
Arginine	1.000 ± 0.025	0.958 ± 0.036	**1.177 ± 0.002**
Aspartate	1.000 ± 0.032	**1.467 ± 0.130**	**5.116 ± 0.400**
Alanine, beta	1.000 ± 0.010	1.268 ± 0.133	**0.387 ± 0.059**
GABA	1.000 ± 0.033	**1.644 ± 0.220**	**0.509 ± 0.023**
Glutamate	1.000 ± 0.085	1.303 ± 0.162	**21.428 ± 2.837**
Glutamine	1.000 ± 0.108	**2.445 ± 0.497**	**1.682 ± 0.214**
Glycine	1.000 ± 0.076	**1.516 ± 0.164**	**0.597 ± 0.055**
Histidine	1.000 ± 0.001	**1.904 ± 0.369**	**3.988 ± 0.282**
Isoleucine	1.000 ± 0.048	**1.725 ± 0.169**	1.190 ± 0.139
Methionine	1.000 ± 0.069	1.589 ± 0.284	**3.275 ± 0.150**
Phenylalanine	1.000 ± 0.079	1.626 ± 0.384	0.812 ± 0.114
Proline	1.000 ± 0.110	**1.892 ± 0.318**	**1.800 ± 0.275**
Pyroglutamic acid	1.000 ± 0.086	1.287 ± 0.119	**2.429 ± 0.218**
Serine	1.000 ± 0.016	**3.981 ± 0.398**	**3.375 ± 0.328**
Treonine	1.000 ± 0.025	**2.507 ± 0.246**	**2.846 ± 0.187**
Tryptophan	1.000 ± 0.132	2.043 ± 0.423	**2.062 ± 0.305**
Valine	1.000 ± 0.070	**1.616 ± 0.178**	**0.462 ± 0.055**
**Organic acids**			
Citric acid	1.000 ± 0.087	**1.392 ± 0.088**	**1.991 ± 0.258**
Glyceric acid	1.000 ± 0.019	1.009 ± 0.075	**3.974 ± 1.083**
Malic acid	1.000 ± 0.042	1.068 ± 0.054	**0.548 ± 0.036**
Phosphoric acid	1.000 ± 0.055	0.952 ± 0.034	**1.315 ± 0.047**
Quinic acid, 3-caffeoyl-, cis-	1.000 ± 0.007	1.073 ± 0.034	nd
Quinic acid, 3-caffeoyl-, trans-	1.000 ± 0.029	**0.605 ± 0.143**	**0.316 ± 0.026**
Saccharic acid	1.000 ± 0.041	1.362 ± 0.141	0.963 ± 0.088
Succinic acid	1.000 ± 0.025	**0.496 ± 0.036**	1.065 ± 0.225
Threonic acid	1.000 ± 0.105	0.805 ± 0.104	**1.672 ± 0.145**
**Sugars and Sugars Alcohol**			
Erythritol	nd	nd	1.000 ± 0.061
Fructose	1.000 ± 0.026	1.041 ± 0.024	**1.219 ± 0.010**
Fucose	1.000 ± 0.057	1.228 ± 0.075	**1.241 ± 0.058**
Galactinol	1.000 ± 0.015	nd	**5.007 ± 0.001**
Glucose	1.000 ± 0.035	1.016 ± 0.035	**1.175 ± 0.033**
Maltose	1.000 ± 0.044	**1.401 ± 0.088**	**4.045 ± 0.828**
Myo-inositol	1.000 ± 0.005	**0.605 ± 0.027**	**0.501 ± 0.030**
Raffinose	1.000 ± 0.076	**0.483 ± 0.092**	nd
Rhamnose	1.000 ± 0.039	**1.322 ± 0.077**	**3.220 ± 0.125**
Sucrose	1.000 ± 0.044	**0.707 ± 0.036**	**0.603 ± 0.096**
Trehalose	1.000 ± 0.001	**0.662 ± 0.001**	**0.775 ± 0.090**
Xylose	1.000 ± 0.031	1.086 ± 0.079	**2.496 ± 0.114**
**Sugar phosphates**			
Fructose-6-phosphate	1.000 ± 0.048	0.917 ± 0.034	**0.754 ± 0.046**
Glucose-6-phosphate	1.000 ± 0.040	0.965 ± 0.050	**0.666 ± 0.033**
**Others**			
Putrescine	1.000 ± 0.084	**2.269 ± 0.198**	**2.599 ± 0.274**
Urea	1.000 ± 0.137	1.077 ± 0.108	0.883 ± 0.105

Table S3. Relative metabolite contents in pericarp of immature green fruits of *SlAREB1* transgenic lines. Metabolites were determined as described in Materials and Methods. Data are normalized with respect to the wild type. Values are presented as means ± SE (four individual fruits per line). Values in boldface indicate significantly difference from the wild type ($p \leq 0.05$) as determined by t-test. nd, not detected.

	WT	A3	A4	S2	S3
**Amino acids**					
Alanine	1.000 ± 0.033	**1.479 ± 0.091**	**1.399 ± 0.189**	**4.496 ± 0.001**	**4.546 ± 0.062**
Arginine	1.000 ± 0.025	**0.907 ± 0.009**	0.842 ± 0.077	**1.180 ± 0.099**	1.046 ± 0.033
Aspartate	1.000 ± 0.032	**1.318 ± 0.032**	**1.453 ± 0.178**	**2.844 ± 0.226**	**2.643 ± 0.026**
Alanine, beta	1.000 ± 0.010	**1.856 ± 0.098**	**1.711 ± 0.248**	**10.360 ± 1.788**	**10.793 ± 0.103**
Phenylalanine	1.000 ± 0.079	**2.539 ± 0.057**	**2.163 ± 0.068**	**4.261 ± 0.240**	**10.971 ± 0.212**
GABA	1.000 ± 0.033	1.225 ± 0.037	1.205 ± 0.156	**3.951 ± 0.677**	**5.572 ± 0.256**
Glutamate	1.000 ± 0.085	1.152 ± 0.039	1.306 ± 0.190	**2.212 ± 0.664**	**2.838 ± 0.138**
Glutamine	1.000 ± 0.108	**1.920 ± 0.332**	**3.006 ± 0.856**	**6.062 ± 0.118**	**7.440 ± 1.019**
Glycine	1.000 ± 0.076	**1.850 ± 0.096**	**1.861 ± 0.328**	2.262 ± 0.690	**6.026 ± 0.129**
Histidine	1.000 ± 0.001	0.984 ± 0.205	1.347 ± 0.156	**5.761 ± 0.001**	**14.744 ± 0.999**
Isoleucine	1.000 ± 0.048	**2.121 ± 0.085**	**2.358 ± 0.453**	**6.753 ± 1.804**	**12.430 ± 0.276**
Methionine	1.000 ± 0.069	**1.347 ± 0.026**	1.392 ± 0.305	**3.127 ± 0.279**	**5.384 ± 0.074**
Proline	1.000 ± 0.110	**1.652 ± 0.121**	1.407 ± 0.198	**10.457 ± 3.135**	**14.319 ± 0.228**
Pyroglutamic acid	1.000 ± 0.086	**1.268 ± 0.030**	**1.808 ± 0.052**	**6.481 ± 0.383**	**5.655 ± 0.293**
Serine	1.000 ± 0.016	**1.316 ± 0.038**	**1.561 ± 0.197**	**9.363 ± 1.312**	**8.755 ± 0.128**
Threonine	1.000 ± 0.025	**1.868 ± 0.034**	**2.417 ± 0.517**	**10.915 ± 0.921**	**11.833 ± 0.141**
Tryptophan	1.000 ± 0.132	1.243 ± 0.171	1.544 ± 0.304	**4.372 ± 0.756**	**18.606 ± 1.259**
Valine	1.000 ± 0.070	**1.809 ± 0.122**	**2.100 ± 0.415**	**6.497 ± 1.489**	**9.659 ± 0.123**
**Organic acids**					
Citric acid	1.000 ± 0.087	1.133 ± 0.018	1.290 ± 0.056	**3.055 ± 0.065**	**2.813 ± 0.113**
Glyceric acid	1.000 ± 0.019	1.071 ± 0.063	0.981 ± 0.080	**3.026 ± 0.167**	**4.409 ± 0.068**
Malic acid	1.000 ± 0.042	1.165 ± 0.012	1.141 ± 0.038	**1.547 ± 0.052**	**1.476 ± 0.043**
Phosphoric acid	1.000 ± 0.055	1.003 ± 0.017	1.006 ± 0.026	**1.961 ± 0.030**	**2.151 ± 0.076**
Quinic acid, 3-caffeoyl-, cis-	1.000 ± 0.007	**1.147 ± 0.045**	**1.235 ± 0.054**	0.968 ± 0.122	nd
Quinic acid, 3-caffeoyl-, trans-	1.000 ± 0.029	**1.144 ± 0.040**	**1.438 ± 0.043**	**0.742 ± 0.004**	**0.708 ± 0.015**
Saccharic acid	1.000 ± 0.041	0.902 ± 0.048	0.936 ± 0.043	0.866 ± 0.053	0.752 ± 0.075
Succinic acid	1.000 ± 0.025	**1.282 ± 0.052**	0.937 ± 0.053	**1.981 ± 0.079**	**3.629 ± 0.035**
Threonic acid	1.000 ± 0.105	0.943 ± 0.080	0.779 ± 0.111	**1.730 ± 0.007**	0.856 ± 0.030
**Sugars, sugar phosphates and sugar alcohols**					
Fructose	1.000 ± 0.026	**0.865 ± 0.014**	**0.714 ± 0.031**	**1.171 ± 0.030**	1.011 ± 0.038
Fructose-6-phosphate	1.000 ± 0.048	**0.844 ± 0.013**	1.142 ± 0.087	1.141 ± 0.037	**1.583 ± 0.052**
Fucose	1.000 ± 0.057	1.205 ± 0.110	1.187 ± 0.065	**1.741 ± 0.271**	**4.433 ± 0.152**
Galactinol	1.000 ± 0.015	**1.231 ± 0.022**	1.245 ± 0.208	**2.042 ± 0.137**	**2.230 ± 0.071**
Glucose	1.000 ± 0.035	0.964 ± 0.012	**0.812 ± 0.033**	**1.277 ± 0.044**	1.064 ± 0.033
Glucose-6-phosphate	1.000 ± 0.040	**0.668 ± 0.016**	1.061 ± 0.082	**1.295 ± 0.091**	**2.438 ± 0.080**
Maltose	1.000 ± 0.044	1.005 ± 0.155	1.042 ± 0.069	**1.384 ± 0.141**	**1.520 ± 0.039**
Myo-inositol	1.000 ± 0.005	0.917 ± 0.005	**0.824 ± 0.077**	**1.930 ± 0.099**	**1.595 ± 0.042**
Raffinose	1.000 ± 0.076	**0.460 ± 0.024**	**0.619 ± 0.061**	nd	nd
Rhamnose	1.000 ± 0.039	0.937 ± 0.018	1.123 ± 0.037	**1.599 ± 0.026**	**1.402 ± 0.020**
Sucrose	1.000 ± 0.044	**0.768 ± 0.028**	**0.779 ± 0.055**	**1.239 ± 0.067**	**0.776 ± 0.006**
Xylose	1.000 ± 0.031	**0.787 ± 0.011**	0.963 ± 0.047	**1.160 ± 0.021**	**1.706 ± 0.027**
**Other**					
Putrescine	1.000 ± 0.084	**1.501 ± 0.018**	**1.545 ± 0.217**	**1.900 ± 0.165**	**3.529 ± 0.244**
Urea	1.000 ± 0.137	0.762 ± 0.082	1.706 ± 0.802	0.972 ± 0.061	1.371 ± 0.117

Table S4. Relative metabolite contents in mature green fruits pericarp of *SlAREB1* transgenic lines. Data are normalized with respect to the wild type. Values are presented as means ± SE (four individual fruits per line). Values in boldface indicate significantly difference from the wild type ($p \leq 0.05$) as determined by t-test. nd, not detected.

	WT	A3	A4	S2	S3
**Amino acids**					
Alanine	1.000 ± 0.082	**1.547 ± 0.177**	**1.271 ± 0.038**	**2.183 ± 0.061**	**1.246 ± 0.014**
Arginine	1.000 ± 0.037	0.934 ± 0.021	0.855 ± 0.062	0.996 ± 0.028	**0.629 ± 0.010**
Aspartate	1.000 ± 0.089	**1.620 ± 0.211**	1.059 ± 0.046	**3.983 ± 0.094**	**2.639 ± 0.037**
Alanine, beta	1.000 ± 0.105	**1.569 ± 0.208**	**1.449 ± 0.050**	**7.029 ± 0.208**	**5.159 ± 0.039**
Phenylalanine	1.000 ± 0.236	**2.400 ± 0.323**	**1.803 ± 0.271**	**2.732 ± 0.069**	**5.572 ± 0.145**
GABA	1.000 ± 0.134	1.168 ± 0.217	0.848 ± 0.027	**2.854 ± 0.071**	**2.906 ± 0.027**
Glutamate	1.000 ± 0.124	**1.677 ± 0.283**	1.248 ± 0.115	**2.276 ± 0.150**	**2.562 ± 0.101**
Glutamine	1.000 ± 0.203	1.355 ± 0.270	1.465 ± 0.226	**2.703 ± 0.225**	**3.172 ± 0.354**
Glycine	1.000 ± 0.108	**1.803 ± 0.251**	**1.413 ± 0.041**	**2.778 ± 0.089**	**1.882 ± 0.026**
Histidine	1.000 ± 0.194	**2.368 ± 0.549**	1.109 ± 0.129	**5.393 ± 0.343**	**6.579 ± 0.109**
Isoleucine	1.000 ± 0.098	**2.272 ± 0.294**	**1.796 ± 0.121**	**4.624 ± 0.111**	**6.114 ± 0.100**
Methionine	1.000 ± 0.179	1.542 ± 0.232	1.350 ± 0.116	**2.621 ± 0.069**	**1.675 ± 0.047**
Pyroglutamic acid	1.000 ± 0.092	**1.876 ± 0.357**	**1.624 ± 0.103**	**2.554 ± 0.080**	**3.164 ± 0.019**
Proline	1.000 ± 0.168	1.223 ± 0.190	0.857 ± 0.059	**5.029 ± 0.228**	**1.760 ± 0.041**
Serine	1.000 ± 0.100	**1.271 ± 0.058**	0.731 ± 0.160	**2.924 ± 0.088**	**2.768 ± 0.036**
Threonine	1.000 ± 0.098	**2.344 ± 0.385**	**1.379 ± 0.121**	**5.187 ± 0.141**	**5.812 ± 0.066**
Tryptophan	1.000 ± 0.207	**1.953 ± 0.178**	1.431 ± 0.263	**2.847 ± 0.194**	**7.073 ± 0.435**
Valine	1.000 ± 0.110	**1.875 ± 0.235**	**1.564 ± 0.049**	**3.414 ± 0.145**	**4.768 ± 0.151**
**Organic acids**					
Citric acid	1.000 ± 0.063	1.165 ± 0.056	1.087 ± 0.022	**1.395 ± 0.041**	**1.516 ± 0.020**
Glyceric acid	1.009 ± 0.075	1.481 ± 0.243	1.026 ± 0.013	**1.679 ± 0.073**	**1.990 ± 0.089**
Malic acid	1.000 ± 0.051	1.201 ± 0.122	**1.180 ± 0.025**	**1.289 ± 0.036**	0.836 ± 0.022
Phosphoric acid	1.000 ± 0.036	**1.418 ± 0.091**	**1.269 ± 0.075**	**1.904 ± 0.048**	**2.070 ± 0.018**
Quinic acid, 3-caffeoyl-, cis-	1.000 ± 0.028	0.994 ± 0.045	**1.149 ± 0.035**	nd	nd
Quinic acid, 3-caffeoyl-, trans-	1.000 ± 0.236	1.603 ± 0.203	1.787 ± 0.331	0.764 ± 0.040	0.821 ± 0.017
Saccharic acid	1.000 ± 0.104	1.200 ± 0.104	0.930 ± 0.198	**0.692 ± 0.037**	**0.662 ± 0.027**
Succinic acid	1.000 ± 0.073	**1.472 ± 0.087**	1.444 ± 0.311	**1.647 ± 0.032**	**1.460 ± 0.034**
Threonic acid	1.000 ± 0.129	0.998 ± 0.097	0.897 ± 0.053	**2.437 ± 0.077**	**1.566 ± 0.037**
**Sugars, sugar phosphates and sugar alcohols**					
Fructose	1.000 ± 0.023	**0.841 ± 0.087**	**0.765 ± 0.035**	0.885 ± 0.017	**0.685 ± 0.005**
Fructose-6-phosphate	1.000 ± 0.037	0.961 ± 0.074	0.975 ± 0.086	**1.137 ± 0.035**	**1.213 ± 0.032**
Fucose	1.000 ± 0.061	**1.432 ± 0.146**	1.122 ± 0.145	**1.595 ± 0.034**	**2.761 ± 0.033**
Glucose	1.000 ± 0.034	1.020 ± 0.047	0.923 ± 0.056	1.006 ± 0.024	**0.683 ± 0.019**
Glucose-6-phosphate	1.000 ± 0.052	0.858 ± 0.045	0.840 ± 0.083	1.081 ± 0.038	0.993 ± 0.011
Maltose	1.000 ± 0.063	0.849 ± 0.082	**0.724 ± 0.055**	0.866 ± 0.033	1.059 ± 0.100
Myo-inositol	1.000 ± 0.045	1.048 ± 0.030	1.055 ± 0.094	**2.393 ± 0.073**	0.845 ± 0.013
Raffinose	1.000 ± 0.190	0.677 ± 0.029	1.139 ± 0.010	nd	nd
Rhamnose	1.000 ± 0.058	1.035 ± 0.033	0.980 ± 0.068	1.105 ± 0.025	1.085 ± 0.011
Sucrose	1.000 ± 0.051	**0.579 ± 0.037**	0.731 ± 0.197	0.963 ± 0.051	**0.488 ± 0.020**
Xylose	1.000 ± 0.072	0.831 ± 0.017	0.916 ± 0.060	0.909 ± 0.027	**1.842 ± 0.052**
**Other**					
Putrescine	1.000 ± 0.087	1.150 ± 0.089	0.807 ± 0.054	1.140 ± 0.119	**1.363 ± 0.038**
Urea	1.000 ± 0.100	1.100 ± 0.129	0.772 ± 0.018	0.903 ± 0.057	1.287 ± 0.371

Table S5. Relative metabolite contents in ripe red fruits pericarp of *SlAREB1* transgenic lines. Data are normalized with respect to the wild type. Values are presented as means ± SE (four individual fruits per line). Values in boldface indicate significantly difference from the wild type (p ≤ 0.05) as determined by t-test. nd, not detected.

	WT	A3	A4	S2	S3
**Amino acids**					
Alanine	1.000 ± 0.166	**1.708 ± 0.152**	0.727 ± 0.237	**1.348 ± 0.088**	0.753 ± 0.110
Aspartate	1.000 ± 0.078	**0.387 ± 0.098**	**0.427 ± 0.113**	**2.520 ± 0.158**	**1.975 ± 0.062**
Alanine, beta	1.000 ± 0.152	1.228 ± 0.202	0.604 ± 0.137	**12.651 ± 1.605**	**4.230 ± 0.297**
GABA	1.000 ± 0.045	0.996 ± 0.206	**0.460 ± 0.139**	**7.477 ± 0.554**	**5.811 ± 0.460**
Glutamate	1.000 ± 0.132	**0.232 ± 0.066**	**0.293 ± 0.088**	**2.119 ± 0.204**	**2.381 ± 0.149**
Glutamine	1.000 ± 0.127	**0.363 ± 0.117**	0.966 ± 0.522	**4.306 ± 0.866**	**3.929 ± 0.596**
Glycine	1.000 ± 0.092	1.186 ± 0.281	0.792 ± 0.194	**4.556 ± 0.668**	**1.849 ± 0.214**
Histidine	1.000 ± 0.071	**0.578 ± 0.065**	**0.558 ± 0.041**	**6.744 ± 1.275**	**5.263 ± 0.388**
Isoleucine	1.000 ± 0.117	0.978 ± 0.258	1.221 ± 0.020	**6.321 ± 0.840**	**4.824 ± 0.388**
Methionine	1.000 ± 0.045	**0.510 ± 0.117**	0.673 ± 0.167	**2.351 ± 0.235**	1.156 ± 0.145
Phenylalanine	1.000 ± 0.140	0.628 ± 0.155	0.973 ± 0.190	**4.124 ± 0.502**	**10.473 ± 0.392**
Pyroglutamic acid	1.000 ± 0.090	**0.517 ± 0.083**	**0.727 ± 0.066**	**2.739 ± 0.191**	**3.345 ± 0.361**
Proline	1.000 ± 0.153	1.273 ± 0.288	0.723 ± 0.043	**4.501 ± 0.437**	0.855 ± 0.085
Serine	1.000 ± 0.098	1.256 ± 0.159	1.001 ± 0.019	**5.074 ± 0.526**	**1.827 ± 0.120**
Treonine	1.000 ± 0.066	0.865 ± 0.185	0.783 ± 0.192	**6.115 ± 0.596**	**4.320 ± 0.213**
Tryptophan	1.000 ± 0.148	0.998 ± 0.279	0.983 ± 0.248	**4.977 ± 0.433**	**8.775 ± 1.400**
Valine	1.000 ± 0.119	1.190 ± 0.316	0.972 ± 0.271	**4.361 ± 0.788**	**2.327 ± 0.206**
**Organic acids**					
Citric acid	1.000 ± 0.130	1.271 ± 0.044	1.142 ± 0.082	**2.177 ± 0.106**	**1.705 ± 0.057**
Glyceric acid	1.000 ± 0.272	1.420 ± 0.145	0.884 ± 0.058	**0.532 ± 0.049**	**0.590 ± 0.016**
Malic acid	1.000 ± 0.066	0.954 ± 0.013	1.073 ± 0.062	**1.812 ± 0.122**	1.162 ± 0.215
Phosphoric acid	1.000 ± 0.036	**0.825 ± 0.017**	0.927 ± 0.018	**1.457 ± 0.072**	**1.435 ± 0.022**
Quinic acid, 3-caffeoyl-, trans-	1.000 ± 0.082	**3.215 ± 0.130**	**1.807 ± 0.247**	0.908 ± 0.028	**1.345 ± 0.031**
Saccharic acid	1.000 ± 0.091	0.854 ± 0.089	**1.381 ± 0.120**	**1.307 ± 0.012**	1.525 ± 0.347
Succinic acid	1.000 ± 0.211	**2.643 ± 0.686**	1.114 ± 0.079	1.022 ± 0.069	1.547 ± 0.145
Threonic acid	1.000 ± 0.087	**0.391 ± 0.074**	**0.430 ± 0.081**	**3.004 ± 0.262**	**2.275 ± 0.095**
**Sugars, sugar phosphates and sugar alcohols**					
Erythritol	1.000 ± 0.060	0.959 ± 0.045	0.860 ± 0.031	**1.868 ± 0.147**	**1.286 ± 0.064**
Fructose	1.000 ± 0.008	**0.822 ± 0.043**	**0.811 ± 0.026**	**1.156 ± 0.031**	**0.837 ± 0.046**
Fructose-6-phosphate	1.000 ± 0.061	1.334 ± 0.140	0.980 ± 0.048	**1.649 ± 0.073**	**1.503 ± 0.186**
Fucose	1.000 ± 0.047	0.828 ± 0.081	0.946 ± 0.015	**1.707 ± 0.238**	**2.308 ± 0.185**
Galactinol	1.000 ± 0.001	2.071 ± 0.681	**0.300 ± 0.016**	nd	nd
Glucose	1.000 ± 0.028	**1.105 ± 0.009**	**0.898 ± 0.026**	**1.291 ± 0.004**	0.954 ± 0.015
Glucose-6-phosphate	1.000 ± 0.050	1.251 ± 0.190	0.803 ± 0.047	**1.617 ± 0.111**	**1.378 ± 0.270**
Maltose	1.000 ± 0.205	**3.529 ± 0.191**	0.706 ± 0.064	**1.416 ± 0.122**	**0.488 ± 0.033**
Myo-inositol	1.000 ± 0.060	0.998 ± 0.194	**0.493 ± 0.036**	**2.176 ± 0.102**	1.012 ± 0.012
Rhamnose	1.000 ± 0.039	0.862 ± 0.048	**0.829 ± 0.043**	**1.145 ± 0.028**	**1.275 ± 0.065**
Sucrose	1.000 ± 0.159	0.657 ± 0.041	**0.466 ± 0.068**	**0.592 ± 0.015**	1.279 ± 0.200
Trehalose	1.000 ± 0.039	**1.483 ± 0.035**	0.946 ± 0.063	**1.777 ± 0.273**	0.914 ± 0.052
Xylose	1.000 ± 0.046	**0.870 ± 0.026**	0.898 ± 0.037	0.917 ± 0.045	**1.749 ± 0.054**
**Other**					
Putrescine	1.000 ± 0.105	**1.698 ± 0.413**	1.390 ± 0.314	**1.467 ± 0.138**	1.200 ± 0.091
Urea	1.000 ± 0.119	0.773 ± 0.037	0.735 ± 0.066	0.848 ± 0.025	0.899 ± 0.072

Table S6. Comparison of metabolite content between genotypes and developmental stages using a two-way analysis of variance. Five levels for the genotype factor (wild type, two antisense lines and two OE lines) and three levels for the development factor (IG, MG, RR) were considered. Significant effects are highlighted in red (p ≤ 0.05). Df, degrees of freedom; MS, mean squares.

Metabolite	Effect	df Effect	MS Effect	df Error	MS Error	F value	p-value	
Alanine	Genotype	4	28.367	47	0.324	87.609	0.00000	*
	Development	2	65.862	47	0.324	203.409	0.00000	*
	Interaction	8	20.714	47	0.324	63.974	0.00000	*
Alanine, beta	Genotype	4	1.395	47	0.005	289.215	0.00000	*
	Development	2	0.725	47	0.005	150.292	0.00000	*
	Interaction	8	0.171	47	0.005	35.513	0.00000	*
Arginine	Genotype	4	0.056	34	0.002	31.627	0.00000	*
	Development	2	0.075	34	0.002	42.305	0.00000	*
	Interaction	8	0.019	34	0.002	10.563	0.00000	*
Aspartate	Genotype	4	290.386	47	1.703	170.491	0.00000	*
	Development	2	482.207	47	1.703	283.112	0.00000	*
	Interaction	8	78.257	47	1.703	45.946	0.00000	*
GABA	Genotype	4	136.478	47	0.733	186.195	0.00000	*
	Development	2	32.497	47	0.733	44.336	0.00000	*
	Interaction	8	3.651	47	0.733	4.981	0.00000	*
Citric acid	Genotype	4	28826.620	47	258.964	111.315	0.00000	*
	Development	2	39899.960	47	258.964	154.075	0.00000	*
	Interaction	8	3889.340	47	258.964	15.019	0.00000	*
Fructose	Genotype	4	62437.816	47	1701.038	36.706	0.00000	*
	Development	2	100157.972	47	1701.038	58.880	0.00000	*
	Interaction	8	12070.031	47	1701.038	7.096	0.00000	*
Fructose-6-P	Genotype	4	0.000	47	0.000	14.971	0.00000	*
	Development	2	0.000	47	0.000	6.926	0.00200	*
	Interaction	8	0.000	47	0.000	3.609	0.00200	*
Fucose	Genotype	4	0.126	47	0.001	157.763	0.00000	*
	Development	2	0.004	47	0.001	4.994	0.01100	*
	Interaction	8	0.007	47	0.001	8.681	0.00000	*
Glucose	Genotype	4	52637.006	47	1003.866	52.434	0.00000	*
	Development	2	85832.700	47	1003.866	85.502	0.00000	*
	Interaction	8	13630.500	47	1003.866	13.578	0.00000	*
Glucose-6-P	Genotype	4	0.003	47	0.000	27.985	0.00000	*
	Development	2	0.004	47	0.000	30.946	0.00000	*
	Interaction	8	0.002	47	0.000	16.756	0.00000	*
Glutamate	Genotype	4	0.932	47	0.015	61.932	0.00000	*
	Development	2	4.736	47	0.015	314.626	0.00000	*
	Interaction	8	0.803	47	0.015	53.37	0.00000	*
Glutamine	Genotype	4	1.756	47	0.044	39.84	0.00000	*
	Development	2	0.177	47	0.044	4.027	0.02400	*
	Interaction	8	0.042	47	0.044	0.959	0.47900	
Glyceric acid	Genotype	4	0.002	47	0.000	6.232	0.00000	*
	Development	2	0.015	47	0.000	49.379	0.00000	*
	Interaction	8	0.005	47	0.000	17.949	0.00000	*
Glycine	Genotype	4	3.751	47	0.059	63.743	0.00000	*
	Development	2	4.453	47	0.059	75.662	0.00000	*
	Interaction	8	1.584	47	0.059	26.921	0.00000	*
Histidine	Genotype	4	0.280	44	0.002	135.938	0.00000	*
	Development	2	0.094	44	0.002	45.758	0.00000	*
	Interaction	8	0.032	44	0.002	15.313	0.00000	*
Myo-inositol	Genotype	4	32.080	47	0.230	139.478	0.00000	*
	Development	2	58.742	47	0.230	255.396	0.00000	*
	Interaction	8	4.145	47	0.230	18.023	0.00000	*
Isoleucine	Genotype	4	37.816	47	0.164	230.163	0.00000	*

	Development	2	4.932	47	0.164	30.017	0.00000	*
	Interaction	8	2.297	47	0.164	13.980	0.00000	*
Malate	Genotype	4	722.748	47	42.371	17.058	0.00000	*
	Development	2	5660.194	47	42.371	133.586	0.00000	*
	Interaction	8	304.552	47	42.371	7.188	0.00000	*
Maltose	Genotype	4	0.009	47	0.000	79.736	0.00000	*
	Development	2	0.036	47	0.000	320.149	0.00000	*
	Interaction	8	0.010	47	0.000	89.898	0.00000	*
Methionine	Genotype	4	0.114	47	0.002	45.941	0.00000	*
	Development	2	0.045	47	0.002	17.994	0.00000	*
	Interaction	8	0.033	47	0.002	13.232	0.00000	*
Phenylalanine	Genotype	4	43.159	47	0.108	400.117	0.00000	*
	Development	2	4.064	47	0.108	37.675	0.00000	*
	Interaction	8	0.709	47	0.108	6.568	0.00000	*
Phosphoric acid	Genotype	4	405.464	47	1.803	224.853	0.00000	*
	Development	2	2.624	47	1.803	1.455	0.24400	
	Interaction	8	18.875	47	1.803	10.467	0.00000	*
Proline	Genotype	4	3.045	47	0.030	100.891	0.00000	*
	Development	2	0.861	47	0.030	28.534	0.00000	*
	Interaction	8	1.032	47	0.030	34.185	0.00000	*
Putrescine	Genotype	4	0.002	47	0.000	5.024	0.00200	*
	Development	2	0.008	47	0.000	17.936	0.00000	*
	Interaction	8	0.001	47	0.000	3.094	0.00700	*
Pyroglutamic acid	Genotype	4	27643.465	47	328.044	84.267	0.00000	*
	Development	2	5216.007	47	328.044	15.900	0.00000	*
	Interaction	8	3283.121	47	328.044	10.008	0.00000	*
Rhamnose	Genotype	4	0.007	47	0.000	20.314	0.00000	*
	Development	2	0.172	47	0.000	469.373	0.00000	*
	Interaction	8	0.003	47	0.000	7.851	0.00000	*
Saccharic acid	Genotype	4	0.000	47	0.000	0.351	0.84200	
	Development	2	0.000	47	0.000	4.675	0.01400	*
	Interaction	8	0.000	47	0.000	2.832	0.01200	*
Serine	Genotype	4	147.692	47	0.618	238.939	0.00000	*
	Development	2	29.219	47	0.618	47.272	0.00000	*
	Interaction	8	12.645	47	0.618	20.457	0.00000	*
Succinate	Genotype	4	0.010	47	0.001	17.533	0.00000	*
	Development	2	0.021	47	0.001	38.389	0.00000	*
	Interaction	8	0.008	47	0.001	15.075	0.00000	*
Sucrose	Genotype	4	4.902	47	0.628	7.811	0.00000	*
	Development	2	28.956	47	0.628	46.145	0.00000	*
	Interaction	8	3.570	47	0.628	5.689	0.00000	*
Threonate	Genotype	4	0.042	47	0.000	137.238	0.00000	*
	Development	2	0.053	47	0.000	173.183	0.00000	*
	Interaction	8	0.016	47	0.000	52.085	0.00000	*
Threonine	Genotype	4	10.901	47	0.034	323.868	0.00000	*
	Development	2	0.772	47	0.034	22.937	0.00000	*
	Interaction	8	0.257	47	0.034	7.628	0.00000	*
Tryptophan	Genotype	4	0.225	47	0.003	84.257	0.00000	*
	Development	2	0.004	47	0.003	1.514	0.23100	
	Interaction	8	0.005	47	0.003	1.827	0.09600	
Urea	Genotype	4	0.001	47	0.001	0.791	0.53700	
	Development	2	0.003	47	0.001	3.576	0.03600	*
	Interaction	8	0.001	47	0.001	1.213	0.31200	
Valine	Genotype	4	73.881	47	0.470	157.256	0.00000	*
	Development	2	76.520	47	0.470	162.873	0.00000	*
	Interaction	8	15.680	47	0.470	33.375	0.00000	*
Xylose	Genotype	4	0.316	47	0.003	161.807	0.00000	*
	Development	2	1.082	47	0.003	554.015	0.00000	*
	Interaction	8	0.035	47	0.003	17.674	0.00000	*

Table S7. Identification of statistically different secondary metabolites in SlAREB1 transgenic tomato fruit pericarp with respect to wild type fruit pericarp ($p \leq 0.01$). Compounds were determined by non-targeted metabolic profiling (LC-ESI-MS) in tomato fruit pericarp. The identification of the chemical formula and putative metabolite was performed by the program "Seven Golden Rules“ and combining literature survey and tomato metabolite databases such as MoToDB. m/z: mass/charge of putative metabolite, rt: retention time, [M]: molecular mass, ESI-: negative electrospray

m/z	Aduct	rt (min)	[M] ESI- (Da)	Proposed formula	Proposed [M] (Da)	Deviation (mDa)	Metabolite
224.0664	[M+H-CH4S]+	19.0224	271.0616	$C_{15}H_{12}O_5$	271.0612	0.41	Naringenin / Naringenin chalcone
289.0951	[M+NH4]+	18.9822	271.0616	$C_{15}H_{12}O_5$	271.0617	-0.09	Naringenin / Naringenin chalcone
303.0554	[M+H-C6H10O5]+	15.3606	464.1009	$C_{21}H_{20}O_{12}$	464.0955	5.39	Isoquercitrin / Miricitrin (Miricetin)
303.0573	[M+H-C6H10O5]+	14.5239	464.1007	$C_{21}H_{20}O_{12}$	464.0955	5.20	Isoquercitrin / Miricitrin (Miricetin)
386.1539	[M+NH4]+	23.8236	368.1222	$C_{17}H_{20}O_9$	368.1107	11.50	Feruloylquinic acid
449.1268	[M+H-C6H10O4]+	15.3277	594.1808	$C_{27}H_{30}O_{15}$	594.1585	22.33	Kaempferol-3-O-rutinoside
449.1204	[M+H]+	16.546	448.1100	$C_{21}H_{20}O_{11}$	448.1006	9.39	Astragalin
465.1064	[M+H]+	14.5037	464.1007	$C_{21}H_{20}O_{12}$	464.0955	5.20	Isoquercitrin / Miricitrin
465.1087	[M+H]+	15.3607	464.1009	$C_{21}H_{20}O_{13}$	464.0955	5.39	Isoquercitrin / Miricitrin
595.1722	[M+H]+	15.3492	594.1682	$C_{27}H_{30}O_{15}$	594.1585	9.74	Kaempferol-3-O-rutinoside
611.1646	[M+H]+	15.3456	610.1583	$C_{27}H_{30}O_{16}$	610.1534	4.87	Rutin / Kaempferol-3,7-di-O-glucoside
611.1681	[M+H]+	14.5164	610.1586	$C_{27}H_{30}O_{16}$	610.1534	5.23	Rutin / Kaempferol-3,7-di-O-glucoside
731.1996	[2M+Na]+	9.7196	354.1028	$C_{16}H_{18}O_9$	354.0951	7.73	Chlorogenic acid

Table S8. Relative content of identified secondary metabolites in fruits of *SlAREB1* transgenic lines. Content of compounds identified in Table S8 and as described in Materials and Methods was normalized with respect to the wild type. Values are presented as means ± SE (three individual fruits per line). Values in boldface indicate significantly difference from the wild type ($p \leq 0.05$) as determined by ANOVA and t-test.

Metabolite	Stage	WT	S2	S3
Naringenin / Naringenin chalcone	IG	1.000 ± 0.067	**1.567 ± 0.060**	**1.627 ± 0.023**
Feruloylquinic acid	IG	1.000 ± 0.012	**1.661 ± 0.034**	**1.493 ± 0.069**
Kaempferol-3-O-rutinoside	RR	1.000 ± 0.015	**0.727 ± 0.033**	**0.652 ± 0.010**
Isoquercitrin / Miricitrin	RR	1.000 ± 0.047	**0.364 ± 0.047**	**0.402 ± 0.003**
Rutin / Kaempferol-3,7-di-O-glucoside	RR	1.000 ± 0.057	**0.383 ± 0.017**	**0.321 ± 0.014**
Chlorogenic acid	IG	1.000 ± 0.054	**1.653 ± 0.072**	**2.145 ± 0.080**
Chlorogenic acid	MG	1.000 ± 0.038	**0.482 ± 0.056**	**0.619 ± 0.061**
Chlorogenic acid	RR	1.000 ± 0.034	**0.446 ± 0.018**	**0.654 ± 0.030**
**Metabolite**	**Stage**	**WT**	**A3**	**A4**
Astragalin	MG	1.000 ± 0.084	**1.418 ± 0.060**	**1.746 ± 0.149**

Table S9. Changes in analyte content in red ripe fruits and leaves in the S2 over-expressor line compared to WT. Significant changes ($p < 0.05$) of more than 30% are highlighted in red (up-regulated) or blue (down-regulated)

	MATURE RED FRUIT S2 / WT			LEAF S2 / WT	
Analyte	mean	s.e.		mean	s.e.
Alanine	1.347	0.088		1.014	0.120
Alanine, beta-	12.646	1.605		1.231	0.087
Arginine	1.474	0.100		2.185	0.292
Aspartic acid,	2.520	0.158		0.885	0.105
Citric acid	2.180	0.106		0.786	0.086
Fructose	1.156	0.031		0.690	0.010
Fructose-6-P	1.649	0.073		1.391	0.092
Fucose	1.710	0.295		0.800	0.041
GABA	7.477	0.544		1.095	0.116
Glucose	1.292	0.040		0.350	0.005
Glucose-6-P	1.617	0.111		1.155	0.096
Glutamic acid	2.119	0.204		0.802	0.122
Glutamine	4.305	0.866		1.013	0.155
Glycine	4.555	0.668		0.890	0.181
Histidine	6.744	1.275		0.951	0.087
Inositol, myo-	2.176	0.102		1.402	0.137
Isoleucine	6.321	0.840		1.348	0.153
Malic acid	1.812	0.122		0.390	0.038
Maltose	1.416	0.126		0.982	0.098
Methionine	2.351	0.235		0.961	0.138
Phenylalanine	4.124	0.502		1.344	0.072
Phosphoric acid	1.458	0.072		2.388	0.342
Proline	4.501	0.437		2.263	0.229
Putrescine	1.467	0.138		0.732	0.111
Pyroglutamic acid	2.739	0.191		1.195	0.110
Quinic acid, 3-caffeoyl-, trans-	0.908	0.028		1.133	0.072
Rhamnose, DL-	1.145	0.028		1.242	0.037
Saccharic acid	1.307	0.012		1.796	0.359
Serine	5.074	0.526		0.973	0.128
succinate	1.021	0.069		1.241	0.121
Sucrose	0.595	0.015		1.195	0.135
threonate	3.004	0.262		1.133	0.095
threonine	6.112	0.596		1.874	0.088
Tryptophan	4.977	0.433		1.027	0.086
Urea	0.848	0.025		1.926	0.083
Valine	4.357	0.788		1.105	0.123

Table S10. Presence of ABA-response elements (ABREs) in the promoters of tomato genes associated to carbon and amino acid metabolism. The analysis was performed using the PLACE database (http://www.dna.affrc.go.jp/PLACE/index.html).

Enzyme	Gene Abreviation	Pathway*	Tomato ID	Nº of ABREs
3-Isopropylmalate dehydrogenase	IDM	Leu (B)	SGN-U578187	1
5-methyl-tetrahydro-pteroyl-triglutamate-homocysteine methyltransferase / methionine synthase	MetS1-3	Cys, Met (B)	SGN-U577720	0
Acetolactate synthase	AHASS	Ile,Leu,Val(B)	SGN-U572742	1
Aminomethyltransferase	AMT	Gly, Ser, Thr	SGN-U581864<br>SGN-U579550	0<br>1
Anthranilate synthase	ASA	Trp (B)	SGN-U573187<br>SGN-U565950<br>Solyc06g005980.2	1<br>0<br>4
Anthranilate phosphoribosyltransferase	TRP	Trp (B)	SGN-U575391<br>SGN-U566340	1<br>2
Argininosuccinate synthase	ARS	Arg (B)	SGN-U571375	2
Argininosuccinate lyase	ARL	Arg (B)	SGN-U569657	1
Arginine decarboxylase	ADC	Arg (C)	SGN-U578034<br>SGN-U578741<br>Solyc07g032590.1<br>SGN-U575555	2<br>1<br>1<br>4
Arogenate dehydrogenase	AAT	Tyr (B)	SGN-U567861	1
Arogenate dehydratase or prephenate dehydratase	PD	Tyr (B)	SGN-U573963<br>SGN-U583010<br>SGN-U572934<br>SGN-U562707<br>SGN-U573964	1<br>3<br>0<br>1<br>1
Asp kinase, monofunctional	AK	Lys(B)	SGN-U569440	3
Asp kinase/homo-Ser dehydrogenase	AK/HSDH	Thr (B)	SGN-U578558<br>SGN-U575816<br>SGN-U601085	1<br>2<br>3
Aspartate aminotransferase	AST	Asp (B)	SGN-U579871<br>SGN-U572392<br>SGN-U580738<br>SGN-U567172<br>SGN-U577882<br>SGN-U572600	2<br>0<br>2<br>5<br>0<br>1
Aspartate-ammonia ligase; asparagine synthetase	AsnS/AS1	Asp, Asn	SGN-U580427<br>SGN-U577532<br>SGN-U567407<br>SGN-U579303<br>Solyc06g054370<br>SGN-U567030	2<br>3<br>1<br>4<br>2<br>0
Asp-semialdehyde dehydrogenase	ASD	Thr (B)	SGN-U577135	0
Branched-chain amino acid aminotransferase	BCAT	Ile,Leu,Val (C)	SGN-U569828<br>SGN-U569830<br>SGN-U569829<br>Solyc00g178340.1<br>SGN-U565681<br>SGN-U569952<br>SGN-U569953<br>SGN-U566152<br>SGN-U579476	1<br>2<br>2<br>0<br>2<br>2<br>3<br>3<br>3
Chorismate mutase	CM	Tyr/Phe (B)	SGN-U575627<br>SGN-U585231	0<br>2
Cystathionine g-synthase	CGS	Met (B)	SGN-U584762<br>SGN-U590566<br>Solyc08g008580.2	1<br>0<br>1

Cysteine synthase A (pyridoxal-5'-phosphate-dependent enzyme, beta family protein)	CysS	Cys (B), Met (B)	SGN-U570112<br>SGN-U579281<br>SGN-U574996<br>SGN-U565167<br>SGN-U585413<br>Solyc04g058120.1<br>SGN-U570460<br>SGN-U570111	2<br>0<br>1<br>0<br>2<br>2<br>3<br>2
Diaminopimelate epimerase	DAPE	Lys(B)	SGN-U569940	1
Dihydrodipicolinate reductase	DHDPR	Lys(B)	SGN-U570280<br>SGN-U578917	1<br>1
Dihydrodipicolinate synthase	DHDPS	Lys (B)	SGN-U563416<br>SGN-U563415	3<br>3
Glutamate dehydrogenase	GDH	Asp, Glu, Arg, Pro	SGN-U574592<br>Solyc06g033860.1<br>SGN-U569234<br>Solyc10g078550.1	4<br>1<br>3<br>1
Glutamate synthase NADH	GLT	Glu (B), Asp(C)	SGN-U573931<br>SGN-U602986<br>Solyc08g044270.2	0<br>5<br>10
Glutamine synthetase o glutamate ammonia ligase	GLN	Glu (C), Gln (B)	SGN-U577193	1
Glycine decarboxylase	GDC	Gly(C)	SGN-U580312	0
Homocysteine S-methyltransferase	HMT3	Met (B)	SGN-U582642<br>SGN-U565941	2<br>4
Homo-Ser kinase	HSK	Thr (B)	SGN-U582271	0
Indole-3-glycerol phosphate synthase	IGPS	Trp (B)	SGN-U563660	5
Isopropylmalate synthase	IPMS	Leu (B)	SGN-U580754	0
Ketol acid reductoisomerase	KARI	Ile, Val, Leu (B)	SGN-U572728<br>SGN-U572725	0<br>2
L,L-Diaminopimelate aminotransferase	AGD	Lys(B)	SGN-U585208<br>SGN-U604034	0<br>6
L-asparaginase	ASNASE	Asp(C)	SGN-U588928<br>SGN-U564199<br>SGN-U583152<br>SGN-U603975<br>SGN-U566421	0<br>0<br>4<br>0<br>2
Lys-ketoglutarate reductase / saccharopine dehydrogenase	LKR/SDH	Lys(C)	Solyc07g017670.1	1
Methionine gamma lyase	MGL	Met (C)	SGN-U565646	2
Methylthioalkylmalate synthase or 2-isopropylmalate synthase	MAM	Met (C)	SGN-U580754	0
N-acetyl-gamma-glutamyl-phosphate reductase		Arg (B)	SGN-U562906	1
Orn d-aminotransferase	d-OAT	Pro (B)	SGN-U563984	1
Orn carbamoyltransferase complex	OCT	Pro (B)	SGN-U575137<br>SGN-U575134	2<br>0
Phe-Ala ammonia lyase	PAL	Phe (C)	SGN-U580050<br>SGN-U581452<br>SGN-U590436<br>SGN-U577267<br>Solyc09g007890.1<br>SGN-U581307<br>SGN-U577677<br>Solyc03g036470.1<br>Solyc03g036480.1<br>Solyc03g071870.1<br>Solyc03g078270.1<br>Solyc03g078280.1<br>Solyc10g011920.1	0<br>0<br>1<br>1<br>0<br>2<br>3<br>1<br>0<br>3<br>3<br>3<br>0

Phosphoglycerate dehydrogenase	PHGDH	Ser (B)	SGN-U566348<br>SGN-U601541	1<br>0
Pyrroline-5-carboxylate synthetase	P5CS	Pro (B)	Solyc08g043170	2
Pyrroline-5-carboxylate reductase	P5CR	Pro (B), Glu (B)	SGN-U567857	3
Pro dehydrogenase/Pro oxidase	ERD5, PRODH	Pro (C), Glu (B)	SGN-U581540<br>SGN-U578070	2<br>3
S-Adenosyl-Met synthetase	SAMS 1-4	Met (C)	SGN-U580783<br>SGN-U580893<br>SGN-U577991	3<br>3<br>3
Serine hydroxymethyltransferase or glycine hydroxymethyltransferase	SHMT	Ser, Gly, Lys (C)	SGN-U575015<br>SGN-U581543<br>SGN-U578810<br>SGN-U580665<br>SGN-U565354<br>SGN-U565355<br>SGN-U577665	0<br>0<br>3<br>2<br>1<br>0<br>4
Serine O-acetyltransferase 2;1	SERAT2;1	Cys, Met (B)	SGN-U573965<br>SGN-U582407<br>SGN-U582417	0<br>0<br>1
Threonine aldolase	THA1	Thr (C)	SGN-U583452	0
Threonine synthase	TS	Thr (B)	SGN-U573081<br>SGN-U573082	3<br>0
Threonine deaminase	TD	Thr (C), Ile (B)	SGN-U570558	1
Tryptophan synthase a	TSA	Trp (B)	SGN-U576881	2
Tryptophan synthase b	TSB	Trp (B)	SGN-U581112<br>SGN-U585350<br>SGN-U580593<br>SGN-U603314	3<br>1<br>2<br>0
Tyrosine aminotransferase	TAT	Tyr (C)	SGN-U566233<br>SGN-U577103<br>Solyc10g008200.2<br>SGN-U563404	0<br>1<br>3<br>1

* (B) = biosynthesis; (C) = catabolism
