# Supplementary material for: The transcription factor AREB1 regulates primary metabolic pathways in tomato fruits
Source: J Exp Bot. 2014 Mar 22;65(9):2351–63. doi: 10.1093/jxb/eru114 (PMC4036503; doi:10.1093/jxb/eru114)
Supplement: Supplementary Data [file supp_eru114_jexbot112896_file002.pdf]

The transcription factor SlAREB1 regulates primary metabolic pathways in tomato fruits

Bastías A, Yañez M, Osorio S, Arbona V, Gómez-Cadenas A, Fernie AR, Casaretto JA

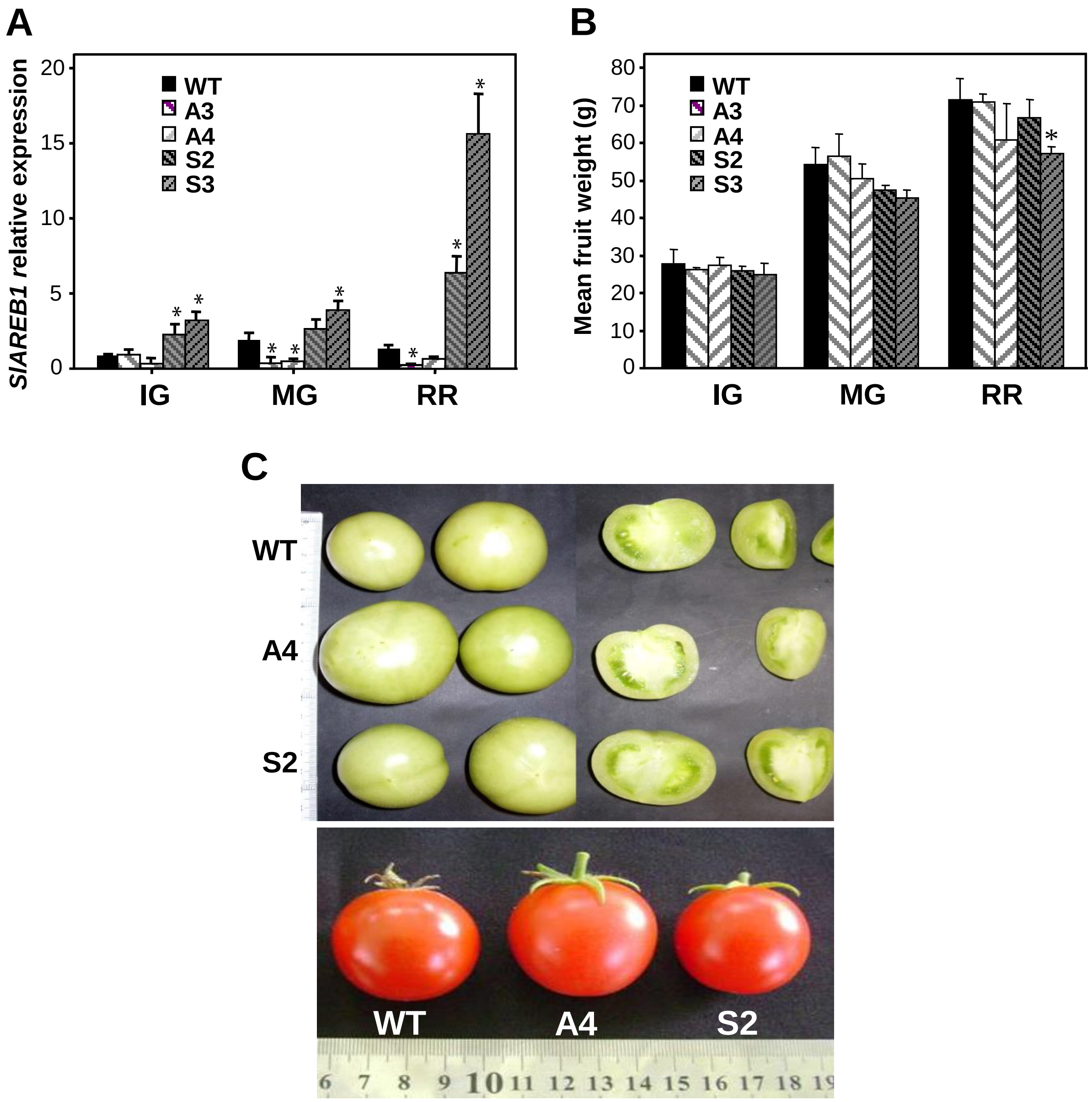

Fig. S1. Characteristics of SlAREB1 transgenic fruits. (A) Transcript levels of *SlAREB1* in pericarp of immature green (IG), mature green (MG) and red ripe fruits (RR) of wild type plants (WT), antisense (A3, A4) and over-expressing (S2, S3) lines quantified by qRT-PCR. Bars indicate mean relative expression ± SE (n=3). (B) Average weight of the fruits from the same lines. (C) Samples of fruits at the mature green and red ripe stages. Asterisks in (A) and (B) indicate significant differences in transcript abundance compared with WT ($P \leq 0.05$).

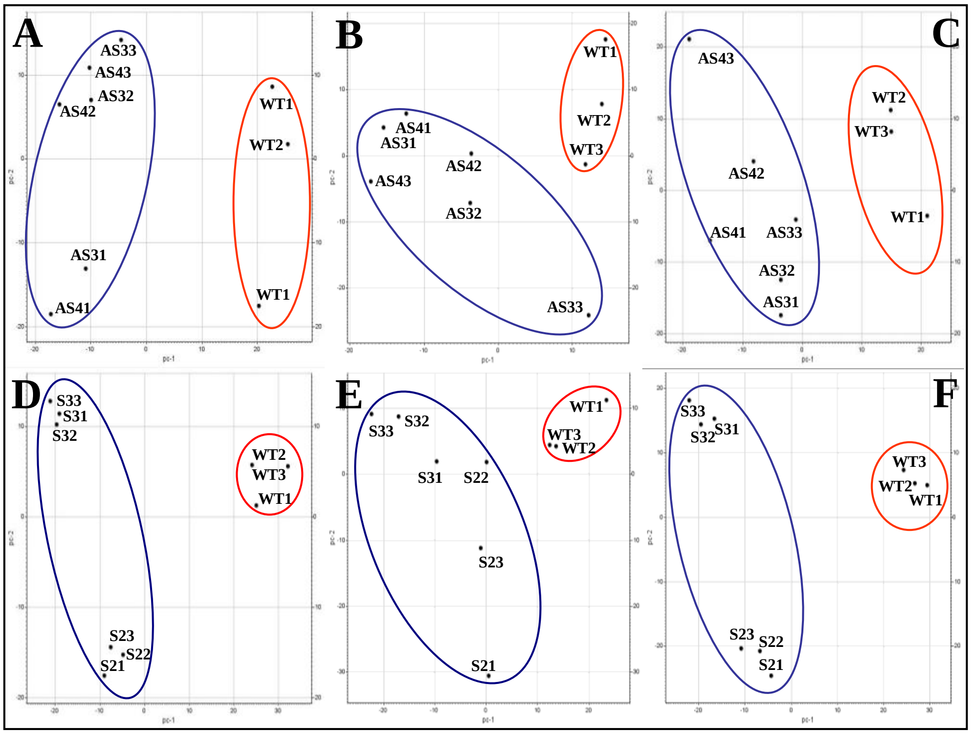

Fig. S2. Differences between metabolic profiles of WT and SlAREB1 transgenic fruit pericarp identified by principal components analysis (PCA) of LC-MS data. PCA of metabolic profiles obtained by LC-MS analysis. The transgenic lines and WT pericarp are grouped in blue and red circles, respectively. **A**, **B** and **C** shown the immature green (IG), mature green (MG) and red ripe (RR) fruit pericarp of WT and *SlAREB1* antisense lines (A3 and A4), respectively. **D**, **E** and **F** depict IG, MG and RR fruit pericarp of WT and *SlAREB1*-overexpressing lines (S2 and S3), respectively. The percentage of the total variance explained by the two principal components varies between 52 and 69%.

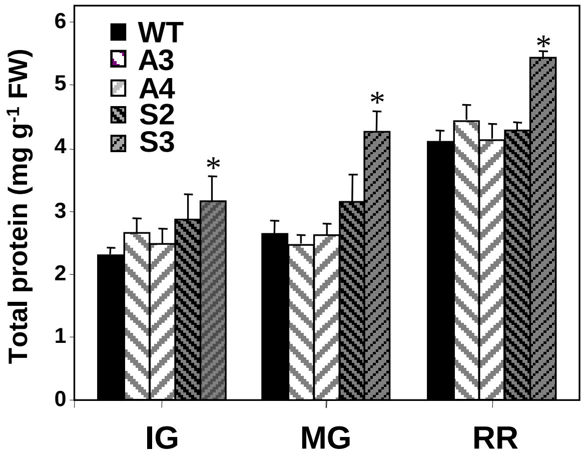

Fig. S3. Total protein content in pericarp of *SlAREB1* transgenic tomato fruits. Total protein content was determined in pericarp of immature green (IG), mature green (MG) and red ripe (RR) fruits of wild type (WT), antisense (A3, A4) and over-expression (S2, S3) lines. Values shown are means ± SE (n=4). Asterisks indicate significant differences with the WT ($p \leq 0.05$).

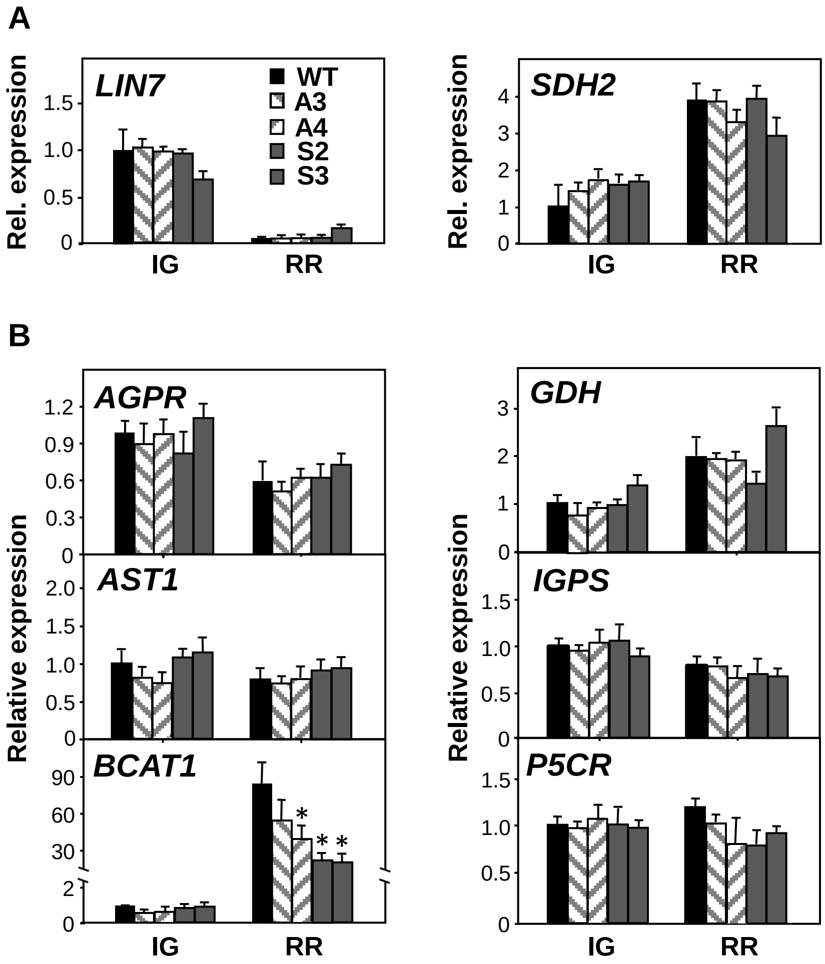

Fig. S4. Expression of other genes associated with (A) sugar and (B) amino acid metabolism in WT and transgenic *SlAREB1* fruits. qRT-PCR analysis of genes encoding the tomato cell wall invertase *LIN7*, succinate dehydronease 2 (*SDH2*), acetyl-gamma-glutamyl-phosphate reductase (*AGPR*), aspartate amino transferase (*AST1*), branched-chain aminotransferase 1 (*BCAT1*), glutamate dehydrogenase (*GDH*), indole-3-glycerol phosphate synthase (IGPS) and pyrroline-5-carboxylate reductase (*P5CR*) in pericarp of immature green (IG) and ripe red (RR) fruits of wild type (WT), AS (A3, A4, light hatched bars) and OE (S2, S3, grey bars) lines. Bars indicate mean relative expression ± SE (n=3) normalized against *ELF1a*. Asterisks indicate significant differences in transcript abundance compared with WT ($p \leq 0.05$).

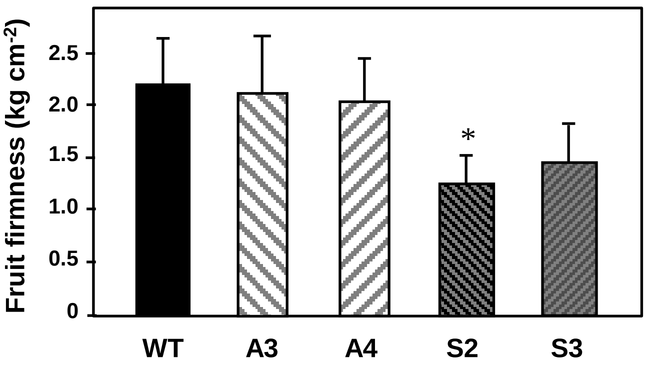

Fig. S5. Firmness of *SlAREB1* transgenic tomato fruits. Fruit firmness was determined by measuring compression using a penetrometer with a 8 mm diameter probe to a vertical depth of 1 mm on opposite sides of full ripen fruits from wild type (WT), antisense (A3, A4) and over-expression (S2, S3) lines. Bars represent means ± SE of 6 full ripen fruits. Asterisks indicate significant differences with the WT ($p \leq 0.05$).
